# Advancing mental health equality: a mapping review of interventions, economic evaluations and barriers and facilitators

**DOI:** 10.1186/s13643-020-01333-6

**Published:** 2020-05-26

**Authors:** Laura-Louise Arundell, Helen Greenwood, Helen Baldwin, Eleanor Kotas, Shubulade Smith, Kasia Trojanowska, Chris Cooper

**Affiliations:** 1 Department of Clinical, Educational and Health Psychology, University College London, London, WC1E 7HB UK; 2 National Collaborating Centre for Mental Health (NCCMH), Royal College of Psychiatrists, 21 Prescot Street, London, E1 8BB UK; 3 York Economics Consortium, University of York, Heslington, York, YO10 5DD UK; 4 Institute of Psychiatry, Psychology and Neuroscience, King’s College London, De Crespigny Park, London, SE5 8AF UK; 5 Nuffield Department of Population Health, University of Oxford, Oxford, OX3 7LF UK

**Keywords:** Mental health, Inequalities, Equality, Mapping review, Health economics, Qualitative research

## Abstract

**Background:**

This work aimed to identify studies of interventions seeking to address mental health inequalities, studies assessing the economic impact of such interventions and factors which act as barriers and those that can facilitate interventions to address inequalities in mental health care.

**Methods:**

A systematic mapping method was chosen. Studies were included if they: (1) focused on a population with: (a) mental health disorders, (b) protected or other characteristics putting them at risk of experiencing mental health inequalities; (2) addressed an intervention focused on addressing mental health inequalities; and (3) met criteria for one or more of three research questions: (i) primary research studies (any study design) or systematic reviews reporting effectiveness findings for an intervention or interventions, (ii) studies reporting economic evaluation findings, (iii) primary research studies (any study design) or systematic reviews identifying or describing, potential barriers or facilitators to interventions.

A bibliographic search of MEDLINE, HMIC, ASSIA, Social Policy & Practice, Sociological Abstracts, Social Services Abstracts and PsycINFO spanned January 2008 to December 2018.

Study selection was performed according to inclusion criteria. Data were extracted and tabulated to map studies and summarise published research on mental health inequalities. A visual representation of the mapping review (a mapping diagram) is included.

**Results:**

Overall, 128 studies met inclusion criteria: 115 primary studies and 13 systematic reviews. Of those, 94 looked at interventions, 6 at cost-effectiveness and 36 at barriers and facilitators. An existing taxonomy of disparities interventions was used and modified to categorise interventions by type and strategy. Most of the identified interventions focused on addressing socioeconomic factors, race disparities and age-related issues. The most frequently used intervention strategy was providing psychological support. Barriers and associated facilitators were categorised into groups including (not limited to) access to care, communication issues and financial constraints.

**Conclusions:**

The mapping review was useful in assessing the spread of literature and identifying highly researched areas versus prominent gaps. The findings are useful for clinicians, commissioners and service providers seeking to understand strategies to support the advancement of mental health equality for different populations and could be used to inform further research and support local decision-making.

**Systematic review registration:**

Not applicable.

## Background

Profound inequalities exist in the access to, experience and outcomes of mental health support for many marginalised or minority communities in the UK [1, 2]. While a number of these characteristics are legally protected by the Equality Act 2010 (race, gender and sexual orientation) [3], and despite National Health Service (NHS) commissioners being statutorily bound by the Health and Social Care Act 2012 [4] to reduce health inequalities, inequities persist, particularly within mental health care where individuals may face an additional level of stigmatisation or discrimination [5]. Accumulating evidence has also established unsatisfactory experiences and outcomes of mental health care in individuals affected by social determinants of poorer health [6, 7] including, but not limited to, socioeconomic deprivation, homelessness and transitional housing and asylum seeker or refugee status [2, 8, 9]. Furthermore, individuals with more than one of these features or protected characteristics are likely to be at further disadvantage, in line with the theory of intersectionality [10].

To reduce the disadvantage associated with these inequalities, meaningful and effective strategies need to be developed. Furthermore, tackling inequalities in mental health care can significantly benefit the wider economy. Consistent evidence suggests that improving access to, and experience of, mental health care can reduce the economic burden of illness and bring long-term cost savings associated with enhanced employability, fewer lost work hours and reduced utilisation of costly health services [11]. To truly bring about effective change and improve outcomes, innovative practice is required at all layers of care to promote cultural, structural and attitudinal shifts.

The NHS has committed to prioritising the reduction of health inequalities in both the *Five Year Forward View for Mental Health* [12] and, more recently, the *NHS Long Term Plan* [13]. Contemporary models of mental health care in high-income countries are primarily based on concepts of mental health and well-being derived from Western culture, and as such, do not necessarily consider cultural and social diversity [14]. Therefore, one way to advance mental health equality may be to commission, or improve uptake of, evidence-based interventions specifically targeted at communities that face inequalities. There is a growing literature base of ‘disparity interventions’ which aim to adapt existing interventions in such communities or develop new interventions tailored to the community of interest. To bring about change, it is important to understand what interventions have been attempted, their effects and overall costs, as well as barriers and facilitators to uptake and success.

This review is part of a larger piece of work—Advancing Mental Health Equality (AMHE) [15], which was commissioned by NHS England as part of the Mental Health Care Pathways programme. The review aims to map the existing literature on disparity interventions in order to inform the delivery of more effective and culturally appropriate care and, ultimately, advance mental health equality.

## Methods

### Study design: systematic map

Systematic mapping reviews aim to draw together existing studies in a specific topic area and develop an understanding of the available data as well as any potential gaps [16–20]. There is no authoritative methodological guidance on how to conduct a systematic mapping review, such as exists for instance for a systematic review [20]. Researchers have gravitated towards a systematic process of study identification, screening and data extraction [20, 21], so that it is clear how the maps have been created. However, research questions are commonly broader in mapping reviews than in systematic reviews [22], study quality is not appraised or graded, and data are presented in a tabular or visual format rather than analysed or fully synthesised [16, 17, 20, 21].

Systematic mapping was chosen as the method for this work since we aimed to understand the studies and data available with a view to establishing further research priorities. The maps are used to consolidate studies in the broad research area of interventions to address mental health inequalities experienced by marginalised or minority communities.

### Research questions and objectives

The research questions were as follows:

1. What studies are there on interventions to address or reduce mental health inequalities?

The objective of this research question was to identify the existing interventions which seek to address mental health inequalities.

2. What are the data from economic evaluations for interventions to address or reduce inequalities in mental health care?

The objective here was to identify studies that assess the economic impact of interventions to address inequalities in mental health care.

3. What are the barriers and facilitators to interventions to address or reduce mental health inequalities?

The objective of the third question was to identify factors which act as barriers and those that can facilitate interventions which seek to address mental health inequalities.

To develop the research questions, we worked with stakeholders and methodologists [16], scoped the literature and informally reviewed the evidence base to identify relevant studies [23]. The purpose was to determine suitable research questions and use these to develop the approach to study identification, as well as pilot inclusion criteria and data extraction for the mapping review [23, 24]. We convened a group of stakeholders to inform the development of this review as part of the AMHE resource [15] developed at the National Collaborating Centre for Mental Health (NCCMH). Stakeholders included people with lived experience of mental health problems, informal carers and people with one or more of the characteristics outlined in Table 1. We also consulted experts in the field of mental health and equalities research; mental health care professionals, including psychiatrists, mental health nurses, approved mental health professionals (AMHPs); and staff working in equality lead roles in the NHS. They contributed to the development of the research questions through a series of focus groups and workshops [15]. They were also integral to the work on the categorisation of barriers. Stakeholders were informed of the progress of the review and had an opportunity to advise further in subsequent meetings and via email.

### Definitions

#### Mental health inequalities and inequities

‘Mental health inequalities’ are defined in this work as differences between population groups in their mental health status and outcomes, including the following:

- the prevention of mental ill health;
- access to and experience of mental health care; and
- outcomes associated with mental ill health.

‘Mental health inequities’ are avoidable inequalities between population groups. They arise from social and material inequalities within society, such as discrimination, stigma and distribution of wealth and resources [25]. This study considers interventions addressing mental health inequities; however, because there is a lack of clear differentiation between definitions of inequity and inequality within the literature, the term ‘mental health inequality’ is used.

#### The population, intervention, outcomes and study designs

##### Population

In this work, the targeted population is defined as people who meet at least one criterion from each of the two types of criteria pertaining to: (a) disorder/problem type AND (b) having one or more specific characteristics.

A. Population: Disorder/problem type

People who have a diagnosis, or who are at risk, of any of the following mental health conditions/disorders or problems:

- anxiety
- bipolar disorder
- antisocial behaviour and conduct disorders (in children and young people)
- depression
- eating disorders
- mental health problems in the pregnancy and postnatal period
- personality disorders
- psychosis and schizophrenia
- self-harm.

This list is derived from the National institute for Health and Care Excellence’s (NICE) categorisation of guidance and pathways for the above specified conditions [26]. We have cross-referenced the list with the International Classification of Diseases’ (ICD-10) [27] classification of mental health disorders for conditions that fall under the categories of:

- F20-29: Schizophrenia, schizotypal and delusional disorders
- F30-39: Mood (affective) disorders
- F40-48: Neurotic, stress-related and somatoform disorders
- F60-69: Disorders of personality and behaviour in adult persons
- F91: Conduct disorders (in: Behavioural and emotional disorders with onset usually occurring in childhood and adolescence)

B. Population: Having specific characteristics
  People who have one or more of the characteristics outlined in equality impact assessments used by NICE in the development of guidelines. These characteristics served as a basis; we broke them down to identify the specific areas that we wished to focus on (see Table 1).

**Table 1:** Population characteristics (protected or other) considered for inclusion in this review

Characteristics	Populations
Protected characteristics (Equality Act 2010)	
Age	Children and young people^a^
	Older adults^a^
Disability	People with intellectual/learning disability and/or autism
	People with physical or sensory impairment
Race	Cultural and ethnic minority groups
Religion or belief	Religious communities
Pregnancy and maternity	New or expectant mothers^a^
Sex	Men or women^a^ People who are intersex^b^
Gender reassignment^e^	People who are transsexual or transgender
Sexual orientation	People with a minority sexual orientation
Other characteristics (from the NICE equality impact assessment)	
Socioeconomic status	People with a low socioeconomic status^c^
Other categories	Other groups in the population who experience poor health because of circumstances often affected by, but going beyond, sharing a protected characteristic or socioeconomic status. The following are examples of groups covered in the NICE guidance [26]: • refugees and asylum seekers • migrant workers • looked after children • homeless people • prisoners and young offenders^d^

^a^Study population with this characteristic must also have an additional characteristic (intersectionality) or need that puts them at risk of experiencing mental health inequalities

^b^This is not explicitly protected by the Equality Act 2010

^c^Depending on policy or other context, this may cover factors such as social exclusion and deprivation associated with geographical areas, or inequalities or variations associated with other geographical distinctions (e.g. rural or urban poverty)

^d^Study population with this characteristic only includes children and young people

^e^This term is that used in the Equality Act 2010 where it is also stated that this term includes the protection of any person who is proposing to undergo or is undergoing a process of changing physiological attributes of biological sex

##### Intervention

In this work, an ‘intervention’ refers to any type of purposeful act, programme, system or deliverable that has been put in place with the intention of addressing or reducing mental health inequality or that is targeted at a specific group at risk of experiencing mental health inequality. These can include treatment interventions, targeted adaptations of existing treatments, policies, intentional organisational or structural changes.

##### Outcomes

This relates to the research questions, such that for research question 1, the outcomes relate to the effectiveness of interventions measured, for example, using relevant clinician- or patient-rated scales (such as symptom severity scales or quality of life measures) or access rates; for research question 2, the outcome is economic in nature, such as a cost-benefit; and for research question 3, the outcome is any actual or perceived barrier or facilitator to intervention uptake and/or success for which themes were extracted.

##### Study design

By research question:

- Research question 1: any primary study evaluating effectiveness
- Research question 2: any economic evaluation
- Research question 3: any primary study evaluating barriers and/or facilitators to intervention uptake.

Systematic reviews were included but they are reported separately to studies identified above and in their own table (see Additional file 3). Editorials, commentaries and letters were not included.

### Search strategy

Study identification (literature search) was undertaken by a qualified information specialist. The following bibliographic databases were systematically searched:

- MEDLINE and MEDLINE In-Process via Ovid
- Health Management Information Consortium (HMIC) via Ovid
- Applied Social Sciences Index Abstracts (ASSIA) via ProQuest
- Social Policy & Practice via Ovid
- Sociological Abstracts via ProQuest
- Social Services Abstracts via ProQuest
- PsycINFO via Ovid.

The full search strategy can be found in Additional file 1 and takes the following form: *(terms for mental health) and (terms for inequalities and reduce) and (terms for economics, meta-analysis, systematic reviews, observational studies, randomised controlled trials (RCTs) and barriers and facilitators)*. The searches were not limited by language and spanned the period from January 2008 to December 2018, a timeframe the authors considered to be within resource limits [28] and agreed with select stakeholders. The search strategies were reviewed by the research team using the PRESS checklist [29]. Resources did not permit the inclusion of the Embase database.

#### Study selection

Studies were double-screened according to the pre-determined inclusion criteria. Title/abstract screening was undertaken using the desktop Rayyan application [30] and resulted in 97.1% agreement. Disagreements were resolved by discussion with the wider review team.

#### Inclusion criteria

For all research questions, to be included in the review the studies had to:

- Focus on a population with:
  - Mental health disorders, conditions or problems that meet the definition for population in this review, and
  - Focus on a population group with protected or other characteristics identified as at risk of experiencing mental health inequalities (see Table 1), and
- Address an intervention, as defined by this review, focused on addressing or reducing mental health inequalities, and
- Meet the following criteria for one or more of the research questions:

- Research question 1: be a primary research study (any study design) or systematic review reporting effectiveness findings for an intervention or interventions
- Research question 2: report the findings of an economic evaluation; include sufficient detail regarding methods and results; the study’s data and results to be extractable (full economic evaluations that compare two or more relevant options and considered both costs and consequences; costing analyses that compared only costs between two or more interventions; and non-comparative studies were all included)
- Research question 3: be a primary research study (any study design) or systematic review identifying and categorising, describing or explaining, potential barriers or facilitators to intervention uptake or success.

#### Data extraction

Where possible, data were extracted from title/abstract, which is consistent with methods of the other mapping reviews [20]. Where study abstracts were insufficient in providing the data required for extraction, lacked clarity or there was any doubt, full texts were retrieved and the relevant data extracted. For primary studies, we extracted study aims, study design, population (sample), population characteristic(s) associated with inequality, intervention details, intervention types and strategies (as applicable), comparator (as applicable) and outcomes. We also extracted currency for primary studies answering research question 2 and outcomes and/or themes for those answering research question 3. For systematic reviews we extracted study aims, included studies, population characteristic(s) associated with inequality, intervention details, intervention types and strategies, comparator(s) (as applicable) and outcomes. For systematic reviews to answer research question 3, we extracted themes pertaining to barriers and facilitators. Data extracted by one researcher were always double-checked by another. The data extraction tables are set out in Additional files 2 & 3.

#### Study quality (risk of bias)

Study quality was not appraised.

## Results

**Fig. 1:**
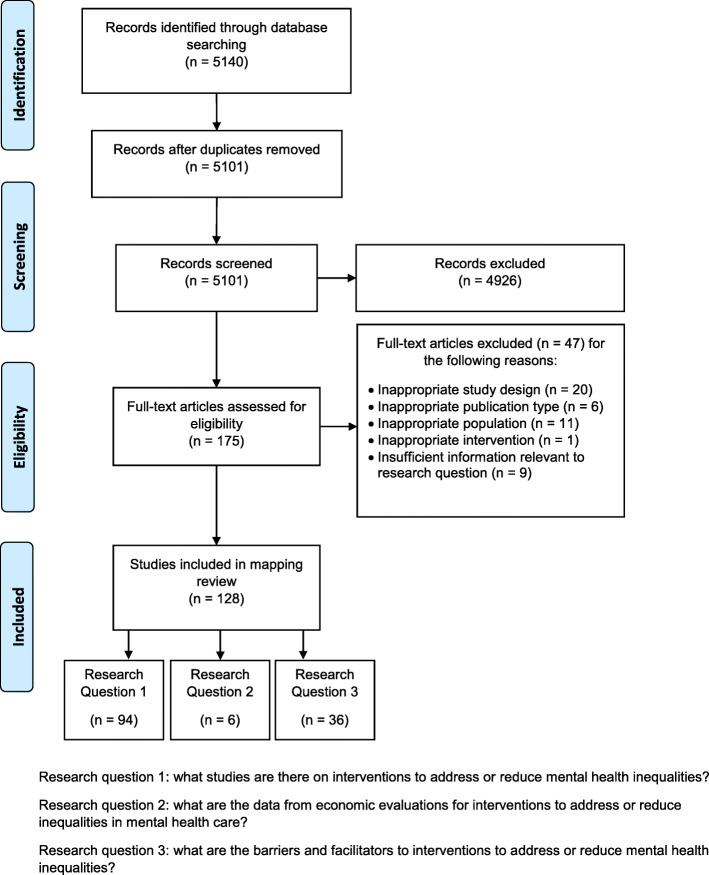
PRISMA flow diagram of research studies search

The overarching findings from the study identification and screening processes are reported to PRISMA reporting guidance; a PRISMA flow chart [31] is included (Fig. 1).

**Fig. 2:**
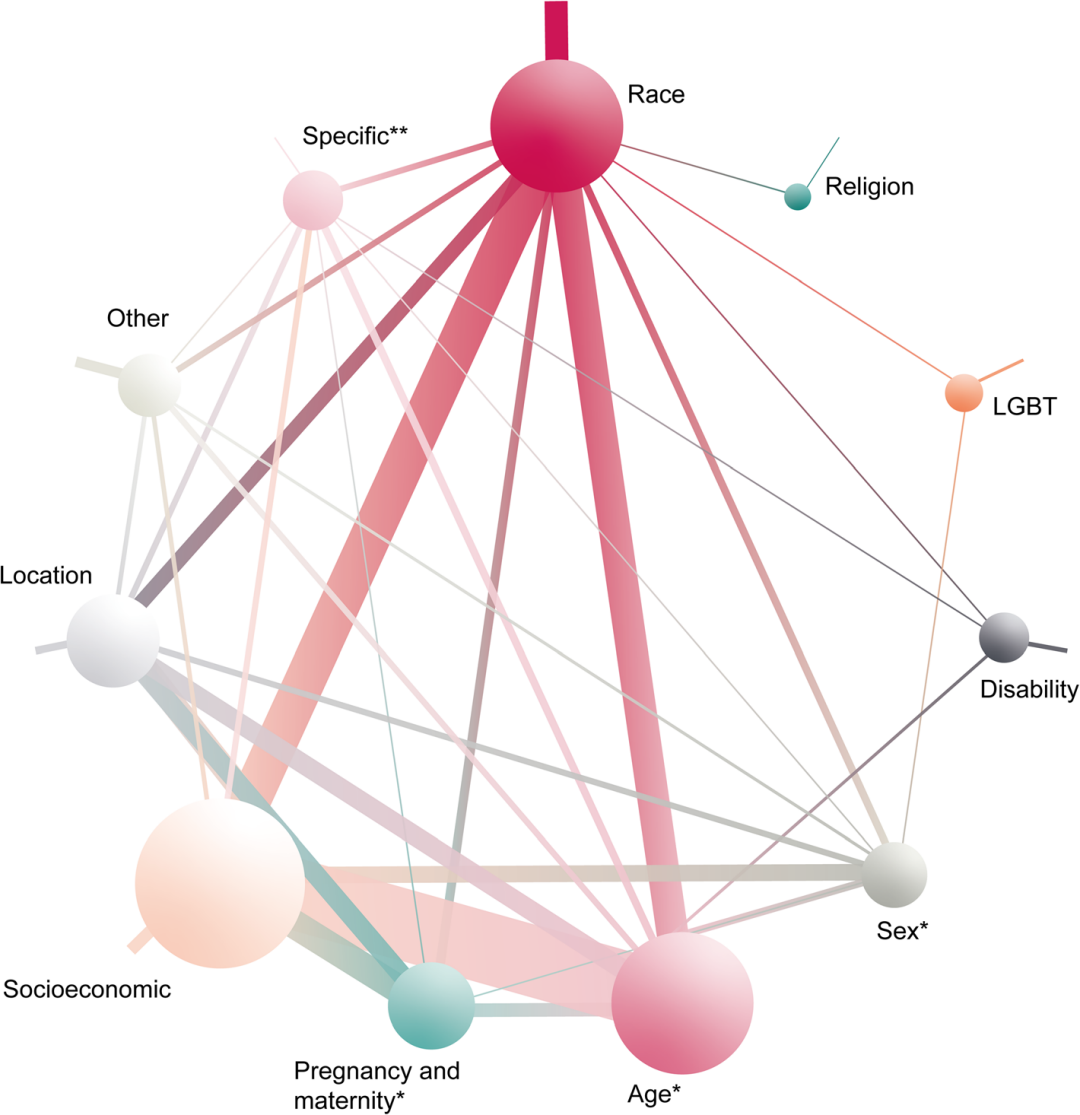
Mapping diagram of primary studies. *Studies examining these characteristics were only included if they also looked at other characteristics. **Specific populations are those who may have a specific set of characteristics and experiences placing them at risk of experiencing mental health inequalities and therefore may already be defined by multiple characteristics (e.g. refugees). Circle size area is asscociated with the number of primary studies that consider a population characteristic. The lines between the circles indicate where characteristics were considered together, while the thickness of the lines indicates the frequency of the association. 49 studies examined 2 population characteristics; the most frequent association was between age and socioeconomic factors. 24 studies examined 3 characteristics, of which age, race and socioeconomic factors were the most frequent associations. 8 studies examined 4 characteristics. 3 studies examined 5 characteristics. 3 studies examined 6 characteristics

The inclusion criteria were met by 128 studies; 115 were primary studies [32–146] and 13 were systematic reviews [147–159]. Relevant data were extracted and tabulated to map the studies (additional file 2); separate tables summarised relevant systematic reviews (see Table 2 and additional file 3). Maps were used to consolidate primary studies that addressed the broad research area of mental health inequalities. Using Adobe Illustrator [160], we also developed a visual representation of the mapping review in the form of a mapping diagram (Fig. 2).

**Table 2:** Systematic reviews for research question 1

Study ID	Study intervention details	Intervention type(s)	Intervention strategies employed	Target population(s)
Bhui et al. [149]	Therapeutic communication interventions	Access, intervention	Engaging the community (EC), other—culturally adapted interventions (OCA), other—technology (OT)	Minority ethnicities
Garcia et al. [151]	Collaborative care model for people with limited English proficiency	Access	Restructuring the care team (RSCT), enhancing language, literacy and communication (ELLS), other—culturally adapted interventions (OCA)	People with depression and limited English-speaking proficiency
Gardner et al. [152]	Incredible Years parenting programme	Early intervention, intervention	Delivering education and training (DET), providing psychological support (PPS)	Children aged 2–10 years from socially disadvantaged families (included differential effects for ethnic minorities)
Lucas et al. [154]	Unconditional monetary or financial benefits interventions	Prevention, access	Providing financial incentives or removing financial barriers (PFIP)	Pregnant women and families with children with low socioeconomic status
Pega et al. [155]	Unconditional monetary or financial benefits interventions	Prevention, access	Providing financial incentives or removing financial barriers (PFIP)	Children and adults with low socioeconomic status from low- and middle-income countries
Rojas-Garcia et al. [156]	Psycho-educational interventions for depression	Early intervention, intervention	Delivering education and training (DET), providing psychological support (PPS), other—culturally adapted interventions (OCA)	Minority ethnic mothers with low socioeconomic status
Vallury et al. [157]	Computerised CBT	Access, intervention	Providing psychological support (PPS), other—technology (OT)	People living in rural or remote communities
van der Waerden et al. [158]	Psycho-educational interventions for depression	Early intervention, intervention	Delivering education and training (DET), providing psychological support (PPS), other—culturally adapted interventions (OCA)	Women with low-socioeconomic status
Weaver and Lapidos [159]	Community health workers interventions	Access	Engaging the community (EC), other—culturally adapted interventions (OCA)	Disadvantaged communities including ethnic minorities and immigrants, people of low socioeconomic status, gender (both male and female), pregnancy and location

Intervention types, intervention strategies and target populations are reported

*CBT* Cognitive–behavioural therapy, *DET d*elivering education and training, *EC* engaging the community, *ELLS* enhancing language/literacy and communication, *OCA* other—culturally adapted interventions, *OT* other—technology, *PFIP* providing financial incentives or removing financial barriers, *PPS* providing psychological support, *RSCT* restructuring the care team

We identified 94 studies that addressed research question 1, 6 that addressed research question 2 and 36 that addressed research question 3; some studies were relevant to more than one question. An existing taxonomy of disparities interventions [161] was used and modified by way of expansion (Table 3), to categorise interventions by type (access, early intervention, intervention, prevention) and strategy (Table 4). Target populations in primary studies were categorised by a range of characteristics and characteristic sub-types (Table 5). It should be noted that characteristic sub-types may or may not be mutually exclusive; therefore, the ‘Number of studies by characteristic’ column in Table 5 does not offer a summative count. The mapping diagram (Fig. 2) presents the findings of the mapping review with reference to the number of primary studies that consider different population characteristics and the frequency with which multiple characteristics are considered together across studies.

**Table 3:** Modifications made to expand the taxonomy of disparities interventions used in this study

Intervention strategy as defined by Clarke et al. [161]	Modification(s)
Enhancing language and literacy services (ELLS)	Broadened to include ‘communication’ as follows: enhancing language, literacy and communication i.e. non-verbal languages (sign language and braille) and accessibility devices such as hearing aids and loops, in addition using interpreters and health literacy screening
Other: - home-based care - increased referrals - patient/provider racial/ethnic concordance - adjust therapy regimen	Broadened to include the following additional strategies in the ‘other’ category: - technology (OT) - community revitalisation (OCR) - culturally adapted interventions (OCA) - not otherwise specified (NOS)
Additional intervention strategies not defined by Clarke et al (2013) [161]	
Improving access to support, care and treatment for mental health problems (IASCT)	New category used in this study to sort strategies that address logistical barriers to accessing mental health support, care and treatment aimed at reaching wider populations or decreasing waitlists. It was added to capture intervention strategies that go beyond the provision of solely psychological therapies. It includes new service models or programmes such as the IAPT programme in the UK.

*ELLS* enhancing language and literacy services/enhancing language, literacy and communication, *IAPT* improving access to psychological therapies, *IASCT* improving access to support care and treatment for mental health problems, *NOS* not otherwise specified, *OT* ‘other’ technology, *OCR* ‘other’ community revitalisation, *OCA* ‘other’ culturally adapted interventions

**Table 4:** Summary of intervention strategies for tackling inequalities identified during research question 1

Intervention strategy	Brief description	Population or inequalities targeted	Number of studies included^a^
Delivering education and training (DET)	Delivering skills-based training/teaching or providing information or tools for self-learning. *May be delivered to a person who has, or is at risk of, a mental health problem (e.g. training to aid self-management of symptoms), to the person’s family or teachers (e.g. training in parenting techniques or behaviour management) or to the health professionals who work with individuals with mental health problems (e.g. competence training).*	• Minority ethnic or immigrant communities, indigenous communities, LGBTQ+ communities, people who have a sensory or physical impairment, people who have a learning disability, females young people, older adults • Pregnancy and maternity, socioeconomic factors, rural or remote localities, urban localities	29
Providing reminders and feedback (PRF)	Providing prompts to promote adherence to the intervention or care programme. *Typically delivered as telephone or text reminders to encourage the participant.*	• Minority ethnic or immigrant communities, young people • Pregnancy and maternity, socioeconomic factors	3
Providing psychological support (PPS)	Delivery of psychological therapies that promote well-being, such as CBT or interpersonal therapy. *May be aimed at a person who has an existing mental health problem (intervention) or is at risk of a mental health problem (prevention), such as during pregnancy.*	• Minority ethnic or immigrant communities, indigenous communities, religious communities, LGBTQ+ communities, people who have a sensory or physical impairment, males, young people, older adults, people experiencing homelessness, refugees • Pregnancy and maternity, socioeconomic factors, rural or remote localities, urban localities	45
Restructuring the care team (RSCT)	The addition of new members to an existing care team, the introduction of a new role to the team or the shifting of duties among the team. *Directed at care teams. Changes may occur at a local level (e.g. within a single service or region) or at a national level as a result of change in policy.*	• Minority ethnic or immigrant communities, indigenous communities, females, young people, older adults • Pregnancy and maternity, socioeconomic factors	13
Engaging the community (EC)	Involving community members or organisations in mental health support or education, to improve engagement. This is best done outside of the health care setting. *May include outreach, co-production, education campaigns or the delivery of care in a community.*	• Minority ethnic or immigrant communities, indigenous communities, religious communities, LGBTQ+ communities, females, young people, older adults, refugees • Pregnancy and maternity, socioeconomic factors, rural or remote localities, urban localities	26
Providing financial incentives or removing financial barriers (PFIP)	Offering free provisions or money, subsidised services or removing financial barriers to accessing care or treatment. *May be delivered via policy change (e.g. national change in health insurance policies) or may be targeted at disadvantaged groups (e.g. renewal or regeneration of deprived housing areas).*	• Minority ethnic or immigrant communities, indigenous communities, females, males, young people • Socioeconomic factors, rural or remote localities, urban localities	7
Improving access to testing and screening (IATS)	Improves the accessibility of testing or screening by addressing logistical, social or financial barriers. *May introduce more routine mental health assessments for specific populations or address issues which can impede access to testing, such as diagnostic overshadowing (e.g. with a comorbid physical condition or learning disability).*	• Minority ethnic or immigrant communities • Socioeconomic factors, urban localities	2
Improving access to support, care and treatment for mental health problems (IASCT)	Addresses logistical barriers to accessing psychological therapies in order to reach a wider population or decrease wait-list durations. *May be national programmes addressing logistical barriers (e.g. lengthy waiting lists and lack of resources) or engagement programmes aimed at reaching underserved communities.*	• Minority ethnic or immigrant communities, indigenous communities, religious communities, females, males, young people, older adults, socioeconomic factors, rural or remote localities, urban localities	8
Enhancing language, literacy and communication (ELLS)	Improving language or communication skills in order to improve engagement or adherence to care. *May be delivered to the individual with a mental health problem to improve accessibility of care (e.g. for those with limited proficiency in the local language or those who have a sensory impairment) or to the health professional to improve therapeutic communication with specific communities.*	• Minority ethnic or immigrant communities, people who have a sensory or physical impairment, older adults • Pregnancy and maternity, socioeconomic factors	3
Other—home-based care (OHBC)	Delivery of healthcare or support in the participant’s home. *Typically involves outreach visits from a healthcare professional or peer support worker to the individual with a mental health problem.*	• Minority ethnic or immigrant communities, young people • Pregnancy and maternity, socioeconomic factors	5
Other—culturally adapted interventions (OCA)	Tailored interventions which work within the cultural context of the recipient and take greater account of their cultural background and experiences. *May include culturally modified versions of well-evidenced therapies (e.g. cognitive behavioural therapy) or interventions developed specifically for the community of interest.*	• Minority ethnic or immigrant communities, indigenous communities, religious communities, LGBTQ+ communities, people who have a sensory or physical impairment, females, males, young people, older adults, refugees • Pregnancy and maternity, socioeconomic factors, rural or remote localities, urban localities	26
Other—technology (OT)	Providing information, skills-based training or therapeutic regimens delivered through the Internet, typically via mobile devices. *Often targeted at communities who face logistical barriers to accessing care, but may also be implemented in healthcare settings to improve information exchange between members of a care team.*	• Minority ethnic or immigrant communities, indigenous communities, young people, older adults • Pregnancy and maternity, socioeconomic factors, rural or remote localities	6
Other—community revitalisation (OCR)	Regeneration or renewal of deprived community areas or poorer socioeconomic localities. *Typically provided through government initiatives, investment or policy change.*	• Minority ethnic or immigrant communities, young people • Socioeconomic factors, urban localities	6
Other—not otherwise specified (O-NOS)	Alternative strategies not otherwise specified. *This included moving people to less deprived or distressed localities.*	• Socioeconomic factors, rural or remote localities	1

*CBT* cognitive behavioural therapy, *DET* delivering education and training, *EC* engaging the community, *ELLS* enhancing language/literacy and communication, *IASCT* improving access to support care and treatment for mental health problems, *IATS* improving access to testing and screening, *LGBTQ+* lesbian, gay, bisexual, transgender and other, *OCA* other—culturally adapted interventions, *OCR* other—community revitalisation, *OHBC* other—home-based care, *O-NOS* other—not otherwise specified, *OT* other—technology; *PFIP* providing financial incentives or removing financial barriers, *PPS*, providing psychological support, *PRF* providing reminders and feedback, *RSCT* restructuring the care team. Text in italics provides further detail about the intervention strategies, including examples where relevant.

^a^Number of included studies by intervention for research question 1 and the populations or inequalities targeted. Some studies may be applied to multiple intervention types

**Table 5:** Populations identified and number of included studies by characteristic across all research questions

Characteristic	Number of studies	Characteristic sub-type	Number of studies
1. Race	49	a) Minority ethnic and immigrants	47
		b) Indigenous communities	6
2. Religion	2	a) Religion	2
3. Sexual orientation and gender identity	4	a) LGBTQ+	4
4. Disability	7	a) Physical or sensory impairment	4
		b) Learning disability	5
5. Sex	12	a) Female	9
		b) Male	4
6. Age	56	a) Young people	49
		b) Older adult	8
7. Pregnancy and maternity	21	a) Pregnancy and maternity (including perinatal and postnatal periods)	21
8. Socioeconomic factors	80	a) Socioeconomic factors	80
9. Location	24	a) Rural/remote	16
		b) Urban	9
10. Specific intersectional groups	10	a) Homeless people	3
		b) Youth offenders	1
		c) Refugees	7
11. Other	10	a) Any other	10

*LGBTQI+* lesbian, gay, bisexual, transgender, queer and others

For research question 1, we identified 94 studies of interventions that aimed to address mental health inequalities: 85 primary studies and 9 systematic reviews. A total of 74 unique interventions were identified across primary studies.

We categorised primary study target populations according to the following characteristics: socioeconomic factors (*n* = 65), age (*n* = 46), race/ethnicity (*n* = 29), location (*n* = 17), pregnancy and maternity (*n* = 14), sex (*n* = 10), ‘specific intersectional groups’ (homeless people, youth offenders and refugees; *n* = 6), ‘other’ (*n* = 6), disability (*n* = 3), sexual orientation and gender identity (lesbian, gay, bisexual, transgender, queer and others (LGBTQ+); *n* = 2) and religion (*n* = 2) (see Table 5). The mapping diagram (Fig. 2) presents the number of primary studies that consider different population characteristics using circle area, e.g. the circle for socioeconomic factors is largest, while the circle for religion is smallest. The lines connecting the circles indicate characteristics considered together, with thickness of the connecting lines showing frequency of that association. Forty-nine studies examined 2 population characteristics; the most frequent association was between age and socioeconomic factors. Twenty-four studies examined 3 characteristics, of which age, race and socioeconomic factors were the most frequent associations.

Countries in which primary studies were conducted were also recorded (Table 6), with the majority conducted in the USA (*n* = 34) and the UK (*n* = 17).

**Table 6:** Countries in which primary studies included in the review were conducted, by research question

Country	Number of studies
Research question 1—Effectiveness of interventions to address inequalities in mental health care	
USA	34
UK	17
Australia	7
Ireland	6
The Netherlands	4
Iran	3
India	2
Austria, Belgium, Canada, China, Colombia, France, Germany, Israel, Norway, Pakistan, Portugal, Spain	1 study per country
Research question 2—Economic evaluations of interventions to address mental health inequalities	
Ireland	2
USA	2
UK	1
Research question 2—Barriers and facilitators to interventions to address mental health inequalities	
UK	10
USA	8
Australia	7
Canada	2
Chile and Colombia, Ethiopia, Ireland, Kenya, Sweden	1 study per country

Intervention strategies used most frequently in primary studies were providing psychological support (*n* = 45), delivering education and training (*n* = 29), engaging the community (*n* = 26) and other—culturally adapted interventions (*n* = 26) (Table 4). The most frequently reported intervention strategy in systematic reviews was other—culturally adapted interventions (*n* = 5) followed by providing psychological support (*n* = 4). As with the primary studies, most of the reviews focused on targeting populations based on socioeconomic factors (*n* = 6). The systematic reviews we identified, including information on intervention types, strategies and target populations, are summarised in Table 2.

For research question 2, only 6 economic evaluations were included (Table 7); these were cost-effectiveness studies of which 3 were also identified in research question 1 [100, 119, 152]. Five studies examined population variables related to socioeconomic factors: 3 studies were of children [100, 108, 152], 1 looked at pregnant women [79] and another at adults eligible for Medicaid in the USA [119]. The last study examined the cost-effectiveness of a health-check intervention for people with learning disabilities [121]. All three cost-effectiveness studies of children with low socioeconomic factors examined the Incredible Years parenting programme*.* All cost-effectiveness studies were conducted in either the USA or Europe.

**Table 7:** Cost-effectiveness studies identified for research question 2

Study ID	Intervention details	Intervention strategies employed
Gardner et al [152]	Incredible Years parenting programme	Providing psychological support (PPS), delivering education and training (DET)
Grote et al. [79]	MOMCare intervention	Providing reminders and feedback (PRF), providing psychological support (PPS), pestructuring the care team (RSCT), other—culturally adapted interventions (OCA)
McGilloway et al. [100]	Incredible Years parenting programme	Providing psychological support (PPS), delivering education and training (DET)
O’Neil et al. [108]	Incredible Years parenting programme	Providing psychological support (PPS), delivering education and training (DET)
Rhodes et al. [119]	Chronic Care Initiative	Improving access to testing and screening (IATS), improving access to support, care and treatment for mental health problems (IASCT), restructuring the care team (RSCT)
Romeo et al. [121]	Health check intervention	Improving access to testing and screening (IATS)

*DET* delivering education and training, *IASCT* improving access to support care and treatment for mental health problems, *IATS* improving access to testing and screening, *OCA* other—culturally adapted interventions, *PPS* providing psychological support, *PRF* providing reminders and feedback, *RSCT* restructuring the care team

For research question 3, we identified barriers and facilitators to interventions aimed at addressing mental health inequalities, including the populations with whom certain interventions are used (Table 8).

**Table 8:** Types of barriers, populations at risk and facilitators as identified in the literature

Types of barriers	Populations at risk of experiencing barrier type	Barriers identified in the literature	Facilitators identified in the literature^a^
Limited treatment options and service limitations	Homeless people, pregnant women with low socioeconomic status	• Institutional challenges, such as time/length of session [32, 49] • Inexperienced or unhelpful staff [50] • Lack of provision of home treatment [50] • Lack of service coordination [34] • Limited treatment options [147] • Long waiting lists and availability of treatment [44, 147] • Lack of adequate discharge planning [56] • The use of specialist services as the ‘default’ [57] • Perceived or actual availability of resources [57] • Inappropriate or limited booking systems [66] • Appointments scheduled during working hours [38] • Perceived difficulties in administering treatment [141]	• Diversity of treatment options (e.g. outreach, home-based care, help over the phone, street clinics) [147] • Collaborative agency approach [32]
Perceived or real discrimination (from staff, family or the community)	Aboriginal communities, ethnic minorities	• Clinician bias [49] • Discrimination towards patients from staff [50] • Racism [33] • Failure to acknowledge non-mainstream concepts of health [33] • Stigma and shame around help-seeking [38, 39, 45, 47, 57, 103, 147] • Sociocultural barriers that may reduce motivation for treatment [147] • Fear of harassment [147] • Attitudinal factors [57] • Cultural naivety, insensitivity and discrimination [103] • Existing social and cultural values or norms concerning gender and traditional family structure [109]	• Staff trained in providing culturally appropriate alternatives to mainstream care [33]
Access to care (including physical access, such as transportation)	People with disabilities (learning or physical), homeless people, people with co-occurring substance use problems, people with low socioeconomic status, people living in rural or remote locations, young people with low socioeconomic status	• Difficulties getting an appointment and contacting health providers [50] • Transport and the physical environment of treatment, access to buildings and facilities [44, 50] • Inappropriate referrals and referral rejections [57] • Geographical location of treatment provision [36, 44] • Need for registration at GP practice in order to be treated [66] • Inappropriate or limited booking systems [66]	• Integration of different services [34] • Reducing transportation barriers through use of mobile health interventions [35] • Provision of services within geographical reach [36] • Services provided in close proximity to where people live [37] • Support for people’s ability to access treatment considering their working conditions [38] • Involvement of family in the person’s care [38] • GP as the first point of contact and with a link to external agencies, collaboration between GPs and other healthcare workers [39] • Convenient location and provision of outreach [32] • Internet-based interventions, as these offer flexibility regarding time and location, low effort, accessibility and (sometimes) anonymity [40] • Widened programme/intervention eligibility (e.g. allowing women who already have a child to participate in the programme) [41]
Financial constraints	Homeless people, people with low socioeconomic status, ethnic minorities	• Financial access to medication [42, 89] • Cost of care and treatment [35, 44, 147] • Inadequate income support [56] • Affordability of technological/digital means as requirements for some treatments (e.g. mobile phones, mobile data 3G/4G) [35, 43] • Lack of health insurance [148] • Lack of childcare provisions [66] • Reduction in spending on health and social care [110] • Housing insecurity [41]	• Removal of financial barriers to prescription medication [42] • Reduce the financial costs associated with data usage by consolidating content onto health apps and minimising the need for online linkages [43] • Provision of free health services and treatment [44] • Provision of affordable services within reach of, and financial support for, families with low socioeconomic status [36] • Subsidies for treatment-related expenses [38]
Communication issues	Ethnic minorities, immigrants and migrants, people with disabilities (learning or physical)	• Availability of accessible information [34, 50, 57] • Difficulty contacting practitioners [50] • Perceived ineligibility for treatment based on communication difficulties [57] • Language barriers/lack of translators [84, 89, 127, 148] • Poor literacy [41, 89] • Problems in communicating, articulating or negotiating problems and needs [41, 153]	• Define and provide specific staff training on communication strategies focused on health needs of the identified population (e.g. migrants) [148] • Meeting the needs of people with low literacy using health apps that provide audio recordings, audio-visual displays and diagrams as well as written information [43]
Awareness of available services	Older people, ethnic minorities	• Reliance on informal supports and poor knowledge about services available [116, 147] • Ignorance about services [57] • Lack of understanding from staff about types of care available and who these are designed for [57] • Lack of education about available services and what treatment entails [44, 57] • Lack of knowledge about the healthcare system and about informal networks of healthcare professionals [148]	• Making campaigns more relevant and effective, use of simpler, more positive language, use of less individualistic language (e.g. ‘me’), respecting different beliefs [45] • Community engagement [46] • Primary care professionals to map community activities [46] • Engaging the local targeted community (including members of the religious community, e.g. the local rabbi )[47]
Trust in services or ‘the system’	People living in rural or remote locations, aboriginal communities, ethnic minorities	• Patient cultural views and/or perceptions of the clinician’s culture [49] • Anxiety and/or lack of confidence in asking for help [50] • Fear of medical services [33, 127] • Confidentiality concerns [40, 147] • Negative past experiences with services [147] • Past experience of punitive or forced mental health care making patients unwilling to take up treatment [57] • Concerns about privacy [66] • Decision to seek help from a traditional or religious healer [36] • Fear of ‘asylums’ [45] • Distrust of social workers and doctors, fear of being asked too many questions, lack of trust in measures to protect confidentiality [45, 116]	• Facilitation of opportunities for disclosure through tele-mental health methods [48] • Building trusting relationships [37]
Appropriateness of available services	Aboriginal communities, ethnic minorities, immigrants, children and young people	• Patient cultural views [49] • Limited culturally appropriate services [34, 116] • Diagnostic overshadowing [57] • Complex comorbidity [62] • Technical ability [43] • Inconsistent methods and application of treatment (e.g. for trans-identifying patients) [65] • Lack of GP training in mental health and/or substance use issues [39] • Failure to provide age-appropriate environments [32]	• Provision of culturally appropriate alternatives to mainstream care [33] • Cultural and linguistic competence of staff; cultural reference points [34] • Developing services that are acceptable to people at risk of disadvantage, such as older people and those from ethnic minorities [35] • Making services ‘holistic’ and ensuring ‘cultural safety’ of primary healthcare services [37] • Providing access to male and female therapists, provision of choice in care and maintaining confidentiality [47]

*GP* general practitioner

^a^We have included the various facilitators reported across studies to answer research question 3

Using the input from the lived experience members of the stakeholder group, we categorised the types of barriers reported in the literature (36 studies) into 8 groups: (1) limited treatment options and service limitations, (2) perceived or real discrimination, (3) access to care, (4) financial constraints, (5) communication issues, (6) awareness of available services, (7) trust in services or ‘the system’, (8) appropriateness of available services. Of the 36 included studies, 34 reported information on barriers in their findings, while only 20 reported on facilitators (Table 8).

## Discussion

### Research question 1: What studies are there on interventions to address or reduce mental health inequalities?

The majority (80%) of primary studies focused on targeting populations based on socioeconomic factors (*n* = 65), age (children and young people as well as older adults; *n* = 46) and race/ethnicity (ethnic minorities and indigenous/aboriginal populations; *n* = 29), indicating that these populations are most frequently targeted in interventions to address mental health inequalities in the published literature. However, it should also be noted that the majority of the included studies were conducted in the USA or the UK, limiting the ability to generalise these findings to other countries. We identified very few primary studies targeting populations on the basis of religious affiliation (*n* = 2), sexual or gender identity and sexual orientation (LGBTQ+; *n* = 2) and disability (*n* = 3), and none of the systematic reviews targeted these populations. These findings warrant further investigation as we are unable to conclude, through use of a mapping review, the reasons for these observations. It is likely that these findings might be indicative of the current state of the literature on availability of interventions for these populations. Further and more focused research on interventions designed for these specific populations is needed.

Identification of intervention strategies used to address inequalities across studies, by frequency, is an interesting finding of this mapping review. However, on its own this finding is of limited use and would be more informative when analysed in conjunction with other research on the effectiveness of these strategies with different populations, to better understand what works for different at-risk groups.

### Research question 2: What are the data from economic evaluations for interventions to address or reduce inequalities in mental health care?

Only 6 economic evaluation studies were identified, limiting our ability to adequately address this research question and form a representative picture of the state of the cost-effectiveness literature regarding interventions aimed at addressing mental health inequalities. Half of the included studies were also analysed in research question 1, where the authors had performed a cost-effectiveness analysis as part of the study.

### Research question 3: What are the barriers and facilitators to interventions to address or reduce mental health inequalities?

The identification of 8 types of barriers in the literature suggests that addressing inequalities in mental health care may be hindered by several factors, other than those that tend to be most commonly discussed, such as access to care [147, 162–165] and service integration [166]. The identification and categorisation of barriers and associated facilitators to interventions aimed at tackling inequalities is, therefore, a useful outcome of this mapping review. An understanding of barriers and facilitators can inform future intervention design, clinical practice, service organisation and methods of care delivery. We were able to identify where interventions are likely to encounter challenges in meeting their aims, while also summarising solutions and potentially helpful guidance around what could work. We were also able to identify the population groups that appear at risk of experiencing barriers. Future research should look more deeply at the effectiveness of facilitating factors in improving equality for people from at-risk population groups.

### Strengths and limitations

This review demonstrates a number of important strengths. First, the protocol was informed by expert opinion with sustained input from expert stakeholders, including those with lived experience of mental health problems and related inequalities. This approach ensured that the direction of the research was both person-centred and reflected the priorities of the target populations. The input from clinicians also ensured the research held practical applicability and could be translated to clinical practice. In addition, the search strategy was broad, incorporating a range of pervasive cultural, social and intersectional inequalities. Similarly, the articles covered a wide geographical range across several continents and were not excluded on the basis of language. This approach is particularly pertinent for health equity research and ensures that geographical inequalities are also represented within the literature base.

We should also acknowledge the caveats. First, while a systematic approach was taken, it is not comparable with a full systematic review as only select databases were chosen; we did not search Embase and we were unable to search grey literature. To address research question 3, the search approach taken used a pragmatic cluster of search terms to focus the study identification on the barriers and facilitators to advancing mental health equality. The aim was to identify relevant studies within a manageable volume of studies to screen. It is possible that some studies have been overlooked by this approach and future work may look at the use of different terms. The timeframe for our literature search was narrowed to 2008–2018. We decided on a decade span between the authors and with input from stakeholders, as it was considered to be within resource limits, following guidance in the Cochrane Handbook [28]. As a result of this approach, it is possible that a relevant body of literature has been missed. In particular, considering the lack of literature pertaining to research question 2, it would be beneficial to conduct a full and focused systematic review in the future, to both provide a more encompassing review of the literature and gain a greater insight into the economic effectiveness of these interventions. Furthermore, as this is a mapping review, we did not assess the quality of each study, nor did we determine the effectiveness of the interventions. As such, the findings report the breadth of the data available, but it would be inappropriate to make any firm recommendations. Data were extracted from title/abstract where possible, with extraction from full text only performed when abstracts either lacked the data required or were unclear. While this method of extraction is both efficient and consistent with other mapping reviews [20], it does carry a risk of potentially relevant information being missed.

A further limitation arises from the problematic nature of categorising communities in the literature. The LGBTQ+ movement represents a diverse range of communities, each with unique needs and social perceptions; for example, the transgender community may face entirely different social issues and experiences of care compared with the lesbian community, and so on. Within the literature, however, these distinct groups were often amalgamated under one overarching label and, as such, it was difficult to separate the findings for lesbian, gay, bisexual, transgender and queer communities. Similarly, the Black, Asian and minority ethnic (BAME) label amalgamates a number of distinct cultures and ethnicities with heterogeneous experiences. Much of the literature did not make any further distinctions, once again limiting our ability to draw distinct conclusions for each component group. This approach limits the practical applicability of academic research concerning these communities and highlights a further pervasive inequality to which they are subjected. Ultimately, this requires a more nuanced, accurate reporting within future academic research involving these communities to ensure care can be tailored to their unique needs.

The stipulation applied to our inclusion criteria regarding population characteristics for age, sex and pregnancy and maternity is potentially problematic. This is because, to be considered eligible for inclusion in the review, studies of these populations needed to include people who have another characteristic or need that puts them at risk of experiencing inequalities. Our reasoning for this decision was to avoid including studies of participants from these groups who did not have an identifiable inequality. For example, studies of children and young people as such may not focus on addressing inequalities but might have been included inappropriately had it not been for the caveat applied here. Still, even though necessary, it is possible that the caveat meant that some potentially relevant studies were excluded.

Based on these limitations, we recommend a full systematic review for each of the characteristic sub-types to gain greater insights into the effectiveness of interventions to tackle mental health inequalities and inform national delivery of care for different population groups.

## Conclusions

The mapping review indicated that the majority of mental health inequality interventions identified in this study focus on addressing socioeconomic factors, race disparities and age-related issues (most of which pertain to children and/or young people). The majority of interventions tend to use providing psychological support and delivering education and training as strategies. The review also identified population groups who may be at risk of experiencing barriers to interventions aimed at addressing inequalities. This knowledge is useful for commissioners and service providers seeking to understand what can be done to support the advancement of mental health equality for different populations. The information gained from the mapping review should be used to inform the direction of further research that could influence local commissioning and service provision.

The mapping review was useful in assessing the spread of literature across sub-topics and identifying the highly researched areas (which include interventions aimed at minority races; addressing socioeconomic factors; and age-related inequality issues) versus the prominent gaps (including interventions aimed at marginalised religious groups; the differing and unique needs of groups within the LGBTQ+ community; and people with disabilities). This map supports the identification of these potential gaps in existing research and assists in setting out future research priorities.

## Supplementary information

**Additional file 1.** Search strategy.

**Additional file 2.** Tabulated study characteristics for included primary studies.

**Additional file 3.** Tabulated study characteristics for included systematic reviews.

## Data Availability

Data sharing is not applicable to this article as no datasets were generated or analysed during the study. Information relevant to this study can be accessed in the additional files.

## Abbreviations

AMHE: Advancing Mental Health Equality
AMHP: Approved mental health professional
BAME: Black, Asian and minority ethnic
CYP: Children and young people
DET: Delivering education and training
EC: Engaging the community
ELLS: Enhancing language, literacy and communication
IAPT: Improving access to psychological therapies
IASCT: Improving access to support, care and treatment for mental health problems
IATS: Improving access to testing and screening
ICD-10: International Classification of Diseases
LGBTQ+: Lesbian, gay, bisexual, transgender, queer and others
NICE: National Institute for Health and Care Excellence
NOS: Not otherwise specified
OCA: Other—culturally adapted interventions
OCR: Other—community revitalisation
OHBC: Other—home-based care
O-NOS: Other—not otherwise specified
OT: Other—technology
PFIP: Providing financial incentives or removing financial barriers
PPS: Providing psychological support
PRF: Providing reminders and feedback
SES: Socioeconomic status
RCT: Randomised controlled trial
RSCT: Restructuring the care team
