# Supplementary material for: Advancing mental health equality: a mapping review of interventions, economic evaluations and barriers and facilitators
Source: Syst Rev. 2020 May 26;9:115. doi: 10.1186/s13643-020-01333-6 (PMC7251669; doi:10.1186/s13643-020-01333-6)
Supplement: Supplementary file 1 — Additional file 1. Search strategy. [file 13643_2020_1333_MOESM1_ESM.docx]

Additional File 1: Search Strategy

| **Databases** | **Date searched** | **No. retrieved** |
| --- | --- | --- |
| MEDLINE (Ovid), Epub ahead of print and MEDLINE In-Process (Ovid) | 13/12/2018 | 3973 |
| ASSIA | 13/12/2018 | 81 |
| HMIC | 13/12/2018 | 208 |
| Social Policy & Practice | 13/12/2018 | 169 |
| Sociological Abstracts and Social Services Abstracts | 13/12/2018 | 61 |
| PsycINFO | 13/12/2018 | 2109 |

| **Database: Medline** |
| --- |
| Strategy used:   \| 1 \| Anxiety/ \| 72329 \| \| --- \| --- \| --- \| \| 2 \| anxiety disorders/ or agoraphobia/ or anxiety, separation/ or neurocirculatory asthenia/ or neurotic disorders/ or obsessive-compulsive disorder/ or hoarding disorder/ or panic disorder/ or phobic disorders/ or phobia, social/ \| 75134 \| \| 3 \| "bipolar and related disorders"/ or bipolar disorder/ \| 37847 \| \| 4 \| Depression/ \| 105329 \| \| 5 \| "feeding and eating disorders"/ or anorexia nervosa/ or binge-eating disorder/ or bulimia nervosa/ or "feeding and eating disorders of childhood"/ or female athlete triad syndrome/ or food addiction/ or night eating syndrome/ or pica/ \| 28350 \| \| 6 \| personality disorders/ or antisocial personality disorder/ or borderline personality disorder/ or compulsive personality disorder/ or dependent personality disorder/ or histrionic personality disorder/ or hysteria/ or paranoid personality disorder/ or passive-aggressive personality disorder/ or schizoid personality disorder/ or schizotypal personality disorder/ \| 39474 \| \| 7 \| Child Behavior Disorders/ \| 19782 \| \| 8 \| mental health/ or exp mental disorders/ \| 1166964 \| \| 9 \| Depression, Postpartum/ \| 4828 \| \| 10 \| self-injurious behavior/ or self mutilation/ or suicide/ or suicidal ideation/ or suicide, assisted/ or suicide, attempted/ \| 65139 \| \| 11 \| (anxiety or anxious or agoraphobia* or neurocirculat* asthenia* or neurotic* or obsessive-compulsive or OCD or hoard* disorder* or panic* disorder* or phobia* or phobic* or bipolar* or depress* or eat* disorder* or anorexia or binge-eat* or bulimia* or female athlete triad or food addict* or night eat* syndrome or pica or hysteria* or mental* health*).tw. \| 703675 \| \| 12 \| (self-injur* behavio?r or self mutilat* or suicide or suicidal or self* harm*).tw. \| 70950 \| \| 13 \| ((personalit* or mental* or Child* Behavio?r or Neurocognit* or Trauma* or Neurotic* or Mood or Disrupt* or Impuls* or Dissociat* or paranoi* psycho*) adj4 disorder*).tw. \| 96716 \| \| 14 \| (Capgras Syndrome* or Delusional Parasitosis or Morgellon* or Schizophren*).tw. \| 114758 \| \| 15 \| 1 or 2 or 3 or 4 or 5 or 6 or 7 or 8 or 9 or 10 or 11 or 12 or 13 or 14 \| 1692409 \| \| 16 \| ((inequal$ or inequit* or equal$ or imbalan$ or disadvant$) and (reduc$ or improv$ or address*)).ti,ab,kw,ot. \| 146013 \| \| 17 \| 15 and 16 \| 11977 \| \| 18 \| comment/ or editorial/ or letter/ \| 1678943 \| \| 19 \| 17 not 18 \| 11904 \| \| 20 \| Economics/ \| 26983 \| \| 21 \| "costs and cost analysis"/ \| 46634 \| \| 22 \| Cost allocation/ \| 1991 \| \| 23 \| Cost-benefit analysis/ \| 74837 \| \| 24 \| Cost control/ \| 21295 \| \| 25 \| Cost savings/ \| 10995 \| \| 26 \| Cost of illness/ \| 24303 \| \| 27 \| Cost sharing/ \| 2383 \| \| 28 \| cost*.mp. \| 626958 \| \| 29 \| (economic$ or pharmacoeconomic$ or price$ or pricing).tw. \| 262432 \| \| 30 \| ((systematic adj review*) or meta analy* or metaanaly*).tw. \| 217455 \| \| 31 \| meta-analysis as topic/ or Meta-Analysis.pt. \| 110705 \| \| 32 \| randomized controlled trial.pt. \| 472946 \| \| 33 \| controlled clinical trial.pt. \| 92795 \| \| 34 \| randomized.ab. \| 429081 \| \| 35 \| placebo.ab. \| 193910 \| \| 36 \| randomly.ab. \| 301901 \| \| 37 \| trial.ab. \| 447575 \| \| 38 \| (barrier* or facilitat* or qualitat*).tw. \| 902588 \| \| 39 \| qualitative research/ \| 42802 \| \| 40 \| Epidemiologic studies/ or exp case control studies/ or exp cohort studies/ or Cross-sectional studies/ \| 2225583 \| \| 41 \| Case control.tw. \| 111971 \| \| 42 \| (cohort adj (study or studies)).tw. \| 166407 \| \| 43 \| Cohort analy$.tw. \| 6624 \| \| 44 \| (Follow up adj (study or studies)).tw. \| 46061 \| \| 45 \| (observational adj (study or studies)).tw. \| 87168 \| \| 46 \| (Longitudinal or Retrospective or Cross sectional).tw. \| 921441 \| \| 47 \| or/20-46 \| 5118621 \| \| 48 \| 19 and 47 \| 6329 \| \| 49 \| limit 48 to yr="2008 -Current" \| 3973 \| |

| **Database: ASSIA** |
| --- |
| Strategy used:  (((MAINSUBJECT.EXACT("Anxiety") OR MAINSUBJECT.EXACT("Separation anxiety") OR MAINSUBJECT.EXACT("Anxiety disorders") OR MAINSUBJECT.EXACT("Agoraphobia") OR MAINSUBJECT.EXACT("Generalized anxiety disorders") OR MAINSUBJECT.EXACT("Social phobia") OR MAINSUBJECT.EXACT("Phobic anxiety") OR MAINSUBJECT.EXACT("Phobias") OR MAINSUBJECT.EXACT("Panic disorders")) OR (MAINSUBJECT.EXACT("Personality disorders") OR MAINSUBJECT.EXACT("Suicide") OR MAINSUBJECT.EXACT("Maternal depression") OR MAINSUBJECT.EXACT("Parasuicide") OR MAINSUBJECT.EXACT("Assisted suicide") OR MAINSUBJECT.EXACT("Dependent personality") OR MAINSUBJECT.EXACT("Bulimia nervosa") OR MAINSUBJECT.EXACT("Pica") OR MAINSUBJECT.EXACT("Postnatal depression") OR MAINSUBJECT.EXACT("Bipolar affective disorder") OR MAINSUBJECT.EXACT("Psychiatric disorders") OR MAINSUBJECT.EXACT("Histrionic personality disorder") OR MAINSUBJECT.EXACT("Selfinjury") OR MAINSUBJECT.EXACT("Anorexia nervosa") OR MAINSUBJECT.EXACT("Parental depression") OR MAINSUBJECT.EXACT("Compulsive eating") OR MAINSUBJECT.EXACT("Paternal depression") OR MAINSUBJECT.EXACT("Depression") OR MAINSUBJECT.EXACT("Childhood depression") OR MAINSUBJECT.EXACT("Mental health") OR MAINSUBJECT.EXACT("Borderline personality disorder") OR MAINSUBJECT.EXACT("Binge eating") OR MAINSUBJECT.EXACT("Hoarding") OR MAINSUBJECT.EXACT("Hyperphagia") OR MAINSUBJECT.EXACT("Schizotypal personality disorders") OR MAINSUBJECT.EXACT("Compulsive behaviour") OR MAINSUBJECT.EXACT("Hysteria") OR MAINSUBJECT.EXACT("Antisocial personality disorder") OR MAINSUBJECT.EXACT("Paranoid schizophrenia") OR MAINSUBJECT.EXACT("Passive-Aggressive personality disorder") OR MAINSUBJECT.EXACT("Eating disorders") OR MAINSUBJECT.EXACT("Dysphagia") OR MAINSUBJECT.EXACT("Paranoid delusion"))) OR (ti,ab((anxiety OR anxious OR agoraphobia* OR neurocirculat* asthenia* OR neurotic* OR obsessive-compulsive OR OCD OR hoard* disorder* OR panic* disorder* OR phobia* OR phobic* OR bipolar* OR depress* OR eat* disorder* OR anorexia OR binge-eat* OR bulimia* OR female athlete triad OR food addict* OR night eat* syndrome OR pica OR hysteria* OR mental* health*)) OR ti((self-injur* behavio?r OR self mutilat* OR suicide OR suicidal OR self* harm*)) OR ti(((personalit* OR mental* OR Child* Behavio?r OR Neurocognit* OR Trauma* OR Neurotic* OR Mood OR Disrupt* OR Impuls* OR Dissociat* OR paranoi* psycho*) NEAR4 disorder*)) OR ti((Capgras Syndrome* OR Delusional Parasitosis OR Morgellon* OR Schizophren*)))) AND ti,ab(((inequal OR inequit* OR equal OR imbalan OR disadvant) AND (reduc OR improv OR address*))) AND ((MAINSUBJECT.EXACT("Cost benefit analysis") OR MAINSUBJECT.EXACT("Cost effective analysis") OR MAINSUBJECT.EXACT("Pricing") OR MAINSUBJECT.EXACT("Cost analysis") OR MAINSUBJECT.EXACT("Prices") OR MAINSUBJECT.EXACT("Cost effectiveness") OR MAINSUBJECT.EXACT("Economics") OR MAINSUBJECT.EXACT("Meta-analysis") OR MAINSUBJECT.EXACT("Cohort analysis") OR MAINSUBJECT.EXACT("Cross-sectional studies") OR MAINSUBJECT.EXACT("Epidemiology") OR MAINSUBJECT.EXACT("Case studies") OR MAINSUBJECT.EXACT("Qualitative research")) OR ti,ab(cost* economic OR pharmacoeconomic OR price OR pricing OR (systematic NEAR/4 review*) OR meta analy* OR metaanaly* OR randomized controlled trial OR controlled clinical trial OR randomized OR placebo OR randomly OR trial OR barrier* OR facilitat* OR qualitat* OR Case control OR (cohort NEAR/4 (study OR studies)) OR Cohort analy OR (Follow up NEAR/4 (study OR studies)) OR (observational NEAR/4 (study OR studies)) OR (Longitudinal OR Retrospective OR Cross sectional))) |

| **Database: HMIC** |
| --- |
| Strategy used:   \| 1 \| Anxiety/ \| 973 \| \| --- \| --- \| --- \| \| 2 \| anxiety disorders/ or agoraphobia/ or anxiety, separation/ or neurocirculatory asthenia/ or neurotic disorders/ or obsessive-compulsive disorder/ or hoarding disorder/ or panic disorder/ or phobic disorders/ or phobia, social/ \| 16 \| \| 3 \| "bipolar and related disorders"/ or bipolar disorder/ \| 133 \| \| 4 \| Depression/ \| 2642 \| \| 5 \| "feeding and eating disorders"/ or anorexia nervosa/ or binge-eating disorder/ or bulimia nervosa/ or "feeding and eating disorders of childhood"/ or female athlete triad syndrome/ or food addiction/ or night eating syndrome/ or pica/ \| 168 \| \| 6 \| personality disorders/ or antisocial personality disorder/ or borderline personality disorder/ or compulsive personality disorder/ or dependent personality disorder/ or histrionic personality disorder/ or hysteria/ or paranoid personality disorder/ or passive-aggressive personality disorder/ or schizoid personality disorder/ or schizotypal personality disorder/ \| 295 \| \| 7 \| Child Behavior Disorders/ \| 0 \| \| 8 \| mental health/ or exp mental disorders/ \| 27391 \| \| 9 \| Postnatal Depression/ \| 203 \| \| 10 \| self-injurious behavior/ or self mutilation/ or suicide/ or suicidal ideation/ or suicide, assisted/ or suicide, attempted/ \| 1654 \| \| 11 \| (anxiety or anxious or agoraphobia* or neurocirculat* asthenia* or neurotic* or obsessive-compulsive or OCD or hoard* disorder* or panic* disorder* or phobia* or phobic* or bipolar* or depress* or eat* disorder* or anorexia or binge-eat* or bulimia* or female athlete triad or food addict* or night eat* syndrome or pica or hysteria* or mental* health*).tw. \| 23921 \| \| 12 \| (self-injur* behavio?r or self mutilat* or suicide or suicidal or self* harm*).tw. \| 2357 \| \| 13 \| ((personalit* or mental* or Child* Behavio?r or Neurocognit* or Trauma* or Neurotic* or Mood or Disrupt* or Impuls* or Dissociat* or paranoi* psycho*) adj4 disorder*).tw. \| 2633 \| \| 14 \| (Capgras Syndrome* or Delusional Parasitosis or Morgellon* or Schizophren*).tw. \| 1221 \| \| 15 \| 1 or 2 or 3 or 4 or 5 or 6 or 7 or 8 or 9 or 10 or 11 or 12 or 13 or 14 \| 41258 \| \| 16 \| ((inequal$ or inequit* or equal$ or imbalan$ or disadvant$) and (reduc$ or improv$ or address*)).ti,ab,kw,ot. \| 6019 \| \| 17 \| 15 and 16 \| 798 \| \| 18 \| comment/ or editorial/ or letter/ \| 18 \| \| 19 \| 17 not 18 \| 798 \| \| 20 \| Economics/ \| 593 \| \| 21 \| "Cost analysis"/ \| 327 \| \| 22 \| Costing/ \| 534 \| \| 23 \| Cost-benefit analysis/ \| 755 \| \| 24 \| Cost control/ \| 631 \| \| 25 \| Cost savings/ \| 437 \| \| 26 \| Cost of illness/ \| 72 \| \| 27 \| Cost sharing/ \| 30 \| \| 28 \| cost*.mp. \| 29699 \| \| 29 \| (economic$ or pharmacoeconomic$ or price$ or pricing).tw. \| 15019 \| \| 30 \| ((systematic adj review*) or meta analy* or metaanaly*).tw. \| 4839 \| \| 31 \| Meta-Analysis.tw. \| 1416 \| \| 32 \| randomized controlled trial.tw. \| 547 \| \| 33 \| controlled clinical trial.tw. \| 80 \| \| 34 \| randomized.ab. \| 701 \| \| 35 \| placebo.ab. \| 734 \| \| 36 \| randomly.ab. \| 2827 \| \| 37 \| trial.ab. \| 5611 \| \| 38 \| (barrier* or facilitat* or qualitat*).tw. \| 18973 \| \| 39 \| qualitative research/ \| 1250 \| \| 40 \| Epidemiologic studies/ or exp case control studies/ or exp cohort studies/ or Cross-sectional studies/ \| 1444 \| \| 41 \| Case control.tw. \| 1408 \| \| 42 \| (cohort adj (study or studies)).tw. \| 3368 \| \| 43 \| Cohort analy$.tw. \| 143 \| \| 44 \| (Follow up adj (study or studies)).tw. \| 638 \| \| 45 \| (observational adj (study or studies)).tw. \| 1430 \| \| 46 \| (Longitudinal or Retrospective or Cross sectional).tw. \| 10717 \| \| 47 \| or/20-46 \| 76991 \| \| 48 \| 19 and 47 \| 377 \| \| 49 \| limit 48 to yr="2008 -Current" \| 208 \| |

| **Database: Social Policy & Practice** |
| --- |
| Strategy used:   \|  \| 1 \| (anxiety or anxious or agoraphobia* or neurocirculat* asthenia* or neurotic* or obsessive-compulsive or OCD or hoard* disorder* or panic* disorder* or phobia* or phobic* or bipolar* or depress* or eat* disorder* or anorexia or binge-eat* or bulimia* or female athlete triad or food addict* or night eat* syndrome or pica or hysteria* or mental* health*).tw. \| 33846 \| \| --- \| --- \| --- \| --- \| \|  \| 2 \| (self-injur* behavio?r or self mutilat* or suicide or suicidal or self* harm*).tw. \| 3420 \| \|  \| 3 \| ((personalit* or mental* or Child* Behavio?r or Neurocognit* or Trauma* or Neurotic* or Mood or Disrupt* or Impuls* or Dissociat* or paranoi* psycho*) adj4 disorder*).tw. \| 3878 \| \|  \| 4 \| (Capgras Syndrome* or Delusional Parasitosis or Morgellon* or Schizophren*).tw. \| 1442 \| \|  \| 5 \| 1 or 2 or 3 or 4 \| 38169 \| \|  \| 6 \| ((inequal$ or inequit* or equal$ or imbalan$ or disadvant$) and (reduc$ or improv$ or address*)).mp. \| 6821 \| \|  \| 7 \| 5 and 6 \| 673 \| \|  \| 8 \| cost*.mp. \| 17993 \| \|  \| 9 \| (economic$ or pharmacoeconomic$ or price$ or pricing).tw. \| 20357 \| \|  \| 10 \| ((systematic adj review*) or meta analy* or metaanaly*).tw. \| 3032 \| \|  \| 11 \| (Meta-Analysis or randomized controlled trial or controlled clinical trial).tw. \| 947 \| \|  \| 12 \| (randomized or placebo or randomly or trial).ab. \| 3444 \| \|  \| 13 \| (barrier* or facilitat* or qualitat*).tw. \| 25257 \| \|  \| 14 \| Case control.tw. \| 215 \| \|  \| 15 \| (cohort adj (study or studies)).tw. \| 842 \| \|  \| 16 \| Cohort analy$.tw. \| 18 \| \|  \| 17 \| (Follow up adj (study or studies)).tw. \| 395 \| \|  \| 18 \| (observational adj (study or studies)).tw. \| 242 \| \|  \| 19 \| (Longitudinal or Retrospective or Cross sectional).tw. \| 7604 \| \|  \| 20 \| or/8-19 \| 70095 \| \|  \| 21 \| 7 and 20 \| 228 \| \|  \| 22 \| limit 21 to yr="2008 -Current" \| 169 \| |

| **Database: Sociological Abstracts and Social Service Abstracts** |
| --- |
| Strategy used:  (((MAINSUBJECT.EXACT("Anxiety") OR MAINSUBJECT.EXACT("Separation anxiety") OR MAINSUBJECT.EXACT("Anxiety disorders") OR MAINSUBJECT.EXACT("Agoraphobia") OR MAINSUBJECT.EXACT("Generalized anxiety disorders") OR MAINSUBJECT.EXACT("Social phobia") OR MAINSUBJECT.EXACT("Phobic anxiety") OR MAINSUBJECT.EXACT("Phobias") OR MAINSUBJECT.EXACT("Panic disorders")) OR (MAINSUBJECT.EXACT("Personality disorders") OR MAINSUBJECT.EXACT("Suicide") OR MAINSUBJECT.EXACT("Maternal depression") OR MAINSUBJECT.EXACT("Parasuicide") OR MAINSUBJECT.EXACT("Assisted suicide") OR MAINSUBJECT.EXACT("Dependent personality") OR MAINSUBJECT.EXACT("Bulimia nervosa") OR MAINSUBJECT.EXACT("Pica") OR MAINSUBJECT.EXACT("Postnatal depression") OR MAINSUBJECT.EXACT("Bipolar affective disorder") OR MAINSUBJECT.EXACT("Psychiatric disorders") OR MAINSUBJECT.EXACT("Histrionic personality disorder") OR MAINSUBJECT.EXACT("Selfinjury") OR MAINSUBJECT.EXACT("Anorexia nervosa") OR MAINSUBJECT.EXACT("Parental depression") OR MAINSUBJECT.EXACT("Compulsive eating") OR MAINSUBJECT.EXACT("Paternal depression") OR MAINSUBJECT.EXACT("Depression") OR MAINSUBJECT.EXACT("Childhood depression") OR MAINSUBJECT.EXACT("Mental health") OR MAINSUBJECT.EXACT("Borderline personality disorder") OR MAINSUBJECT.EXACT("Binge eating") OR MAINSUBJECT.EXACT("Hoarding") OR MAINSUBJECT.EXACT("Hyperphagia") OR MAINSUBJECT.EXACT("Schizotypal personality disorders") OR MAINSUBJECT.EXACT("Compulsive behaviour") OR MAINSUBJECT.EXACT("Hysteria") OR MAINSUBJECT.EXACT("Antisocial personality disorder") OR MAINSUBJECT.EXACT("Paranoid schizophrenia") OR MAINSUBJECT.EXACT("Passive-Aggressive personality disorder") OR MAINSUBJECT.EXACT("Eating disorders") OR MAINSUBJECT.EXACT("Dysphagia") OR MAINSUBJECT.EXACT("Paranoid delusion"))) OR (ti,ab((anxiety OR anxious OR agoraphobia* OR neurocirculat* asthenia* OR neurotic* OR obsessive-compulsive OR OCD OR hoard* disorder* OR panic* disorder* OR phobia* OR phobic* OR bipolar* OR depress* OR eat* disorder* OR anorexia OR binge-eat* OR bulimia* OR female athlete triad OR food addict* OR night eat* syndrome OR pica OR hysteria* OR mental* health*)) OR ti((self-injur* behavio?r OR self mutilat* OR suicide OR suicidal OR self* harm*)) OR ti(((personalit* OR mental* OR Child* Behavio?r OR Neurocognit* OR Trauma* OR Neurotic* OR Mood OR Disrupt* OR Impuls* OR Dissociat* OR paranoi* psycho*) NEAR4 disorder*)) OR ti((Capgras Syndrome* OR Delusional Parasitosis OR Morgellon* OR Schizophren*)))) AND ti,ab(((inequal OR inequit* OR equal OR imbalan OR disadvant) AND (reduc OR improv OR address*))) AND ((MAINSUBJECT.EXACT("Cost benefit analysis") OR MAINSUBJECT.EXACT("Cost effective analysis") OR MAINSUBJECT.EXACT("Pricing") OR MAINSUBJECT.EXACT("Cost analysis") OR MAINSUBJECT.EXACT("Prices") OR MAINSUBJECT.EXACT("Cost effectiveness") OR MAINSUBJECT.EXACT("Economics") OR MAINSUBJECT.EXACT("Meta-analysis") OR MAINSUBJECT.EXACT("Cohort analysis") OR MAINSUBJECT.EXACT("Cross-sectional studies") OR MAINSUBJECT.EXACT("Epidemiology") OR MAINSUBJECT.EXACT("Case studies") OR MAINSUBJECT.EXACT("Qualitative research")) OR ti,ab(cost* economic OR pharmacoeconomic OR price OR pricing OR (systematic NEAR/4 review*) OR meta analy* OR metaanaly* OR randomized controlled trial OR controlled clinical trial OR randomized OR placebo OR randomly OR trial OR barrier* OR facilitat* OR qualitat* OR Case control OR (cohort NEAR/4 (study OR studies)) OR Cohort analy OR (Follow up NEAR/4 (study OR studies)) OR (observational NEAR/4 (study OR studies)) OR (Longitudinal OR Retrospective OR Cross sectional))) |

| **Database: PsycINFO** |
| --- |
| Strategy used:   \| 1 \| Anxiety/ \| 56928 \| \| --- \| --- \| --- \| \| 2 \| anxiety disorders/ or agoraphobia/ or anxiety, separation/ or neurocirculatory asthenia/ or neurotic disorders/ or obsessive-compulsive disorder/ or hoarding disorder/ or panic disorder/ or phobic disorders/ or phobia, social/ \| 36893 \| \| 3 \| "bipolar and related disorders"/ or bipolar disorder/ \| 25315 \| \| 4 \| Depression/ \| 24681 \| \| 5 \| "feeding and eating disorders"/ or anorexia nervosa/ or binge-eating disorder/ or bulimia nervosa/ or "feeding and eating disorders of childhood"/ or female athlete triad syndrome/ or food addiction/ or night eating syndrome/ or pica/ \| 12195 \| \| 6 \| personality disorders/ or antisocial personality disorder/ or borderline personality disorder/ or compulsive personality disorder/ or dependent personality disorder/ or histrionic personality disorder/ or hysteria/ or paranoid personality disorder/ or passive-aggressive personality disorder/ or schizoid personality disorder/ or schizotypal personality disorder/ \| 25945 \| \| 7 \| child psychopathology/ \| 2792 \| \| 8 \| mental health/ or exp mental disorders/ \| 611256 \| \| 9 \| Postpartum Depression/ \| 4319 \| \| 10 \| self-injurious behavior/ or self mutilation/ or suicide/ or suicidal ideation/ or suicide, assisted/ or suicide, attempted/ \| 39214 \| \| 11 \| (anxiety or anxious or agoraphobia* or neurocirculat* asthenia* or neurotic* or obsessive-compulsive or OCD or hoard* disorder* or panic* disorder* or phobia* or phobic* or bipolar* or depress* or eat* disorder* or anorexia or binge-eat* or bulimia* or female athlete triad or food addict* or night eat* syndrome or pica or hysteria* or mental* health*).tw. \| 623686 \| \| 12 \| (self-injur* behavio?r or self mutilat* or suicide or suicidal or self* harm*).tw. \| 62965 \| \| 13 \| ((personalit* or mental* or Child* Behavio?r or Neurocognit* or Trauma* or Neurotic* or Mood or Disrupt* or Impuls* or Dissociat* or paranoi* psycho*) adj4 disorder*).tw. \| 134409 \| \| 14 \| (Capgras Syndrome* or Delusional Parasitosis or Morgellon* or Schizophren*).tw. \| 119492 \| \| 15 \| 1 or 2 or 3 or 4 or 5 or 6 or 7 or 8 or 9 or 10 or 11 or 12 or 13 or 14 \| 982929 \| \| 16 \| ((inequal$ or inequit* or equal$ or imbalan$ or disadvant$) and (reduc$ or improv$ or address*)).tw. \| 37230 \| \| 17 \| 15 and 16 \| 7057 \| \| 18 \| comment/ or editorial/ or letter/ \| 3191 \| \| 19 \| 17 not 18 \| 7057 \| \| 20 \| Economics/ \| 20846 \| \| 21 \| "costs and cost analysis"/ \| 15765 \| \| 22 \| "cost containment"/ or health care economics/ \| 1347 \| \| 23 \| cost*.mp. \| 105566 \| \| 24 \| (economic$ or pharmacoeconomic$ or price$ or pricing).tw. \| 124052 \| \| 25 \| ((systematic adj review*) or meta analy* or metaanaly*).tw. \| 50005 \| \| 26 \| (Meta-Analysis or randomized controlled trial or controlled clinical trial).tw. \| 42507 \| \| 27 \| (randomized or placebo or randomly).ab. \| 145443 \| \| 28 \| (barrier* or facilitat* or qualitat*).tw. \| 345313 \| \| 29 \| qualitative research/ \| 8041 \| \| 30 \| Epidemiologic studies/ or exp case control studies/ or exp cohort studies/ or Cross-sectional studies/ \| 0 \| \| 31 \| Case control.tw. \| 10004 \| \| 32 \| (cohort adj (study or studies)).tw. \| 18636 \| \| 33 \| Cohort analy$.tw. \| 818 \| \| 34 \| (Follow up adj (study or studies)).tw. \| 12388 \| \| 35 \| (observational adj (study or studies)).tw. \| 9095 \| \| 36 \| (Longitudinal or Retrospective or Cross sectional).tw. \| 193086 \| \| 37 \| or/20-36 \| 891219 \| \| 38 \| 19 and 37 \| 2968 \| \| 39 \| limit 38 to yr="2008 -Current" \| 2109 \| |
