# Supplementary material for: Advancing mental health equality: a mapping review of interventions, economic evaluations and barriers and facilitators
Source: Syst Rev. 2020 May 26;9:115. doi: 10.1186/s13643-020-01333-6 (PMC7251669; doi:10.1186/s13643-020-01333-6)
Supplement: Supplementary file 2 — Additional file 2. Tabulated study characteristics for included primary studies. [file 13643_2020_1333_MOESM2_ESM.docx]

Additional File 2: Primary Studies – study characteristics

Research question 1: what studies are there on interventions to address or reduce mental health inequalities?

Research question 2: what are the data from economic evaluations for interventions to address or reduce inequalities in mental health care?

Research question 3: what are the barriers and facilitators to interventions to address or reduce mental health inequalities?

Table 1. Research question 1 primary studies

| **Study ID (first author, year)** | **Study aims** | **Study design** | **Population sample** | **Population characteristic/s associated with inequality** | **Intervention details** | **Intervention types and strategies** | **Comparator** | **Outcomes** |
| --- | --- | --- | --- | --- | --- | --- | --- | --- |
| Adams 2015 | To evaluate the impact of removing a significant financial barrier to prescription medications (drug caps) on existing black-white disparities in antidepressant treatment rates among patients with diabetes and comorbid depression. | Interrupted time series (ITS) to examine changes in the monthly rate of anti-depressant treatment 24 months before and after the transition of dual enrollees from Medicaid to Medicare Part D drug coverage. | 31 US states. Black and white dual enrollees of Medicare and Medicaid - a 5% nationally representative sample. Adults (18+) with a diagnosis of diabetes of which a subset also had Major Depression. | Race: Black-white disparities in antidepressant treatment. | Removal of drug caps in Medicaid/ Medicare plans  Setting -prescriptive settings in the USA. | Type – access  Strategy - PFIP | States with no removal of drug cap. | - Antidepressant treatment use (proportion of patients using and dosage) |
| Barnes 2009 | To evaluate the impact of volunteer support on maternal depression. | Cluster randomised study | 41 Home-Start local schemes in England = 1,007 mothers enrolled, but only 96 received more than one visit. | New mothers who score 9+ on the Social Disadvantage scale. | Informal volunteer home- support  Setting – home-based, UK. | Types – early intervention, prevention  Strategy - EC | Supported case matched controls and no support | - Emergence of maternal depression - Symptoms of depression |
| Bassilios 2016 | To examine the achievements of ATAPS in relation to its stated objectives using a set of indicators that largely drew on data from a minimum data set designed for the evaluation of ATAPS. | Quantitative data analysis of provider-, consumer- and session-level data. | National consumers of Medicare, referred for a mental disorder. | Low SES, rural or remote locations, Aboriginal communities. | Australian Access to Allied Psychological Services (ATAPS) program.  Setting - primary care in Australia. | Types – access, intervention  Strategy – IASCT | Some comparison to secondary analysis of the previous 'Better Care' program. | - Service use (referral numbers, uptake, service user and consumer characteristics) - clinical outcomes (depression (including post-natal depression), anxiety and suicidal ideation measures |
| Bassilios 2010 | To explore the reciprocal impact of the uptake of psychological treatment delivered by the ATAPS initiative | Quantitative analysis (regression) of General Practice-level data sources. | National consumers of Medicare, referred for a mental disorder. | Rural, remote and metropolitan areas. | Australian Access to Allied Psychological Services (ATAPS) program, compared to the Better Care program  Setting – primary care in Australia. | Types – access, intervention  Strategies – IASCT, PFIP | Better Care program | - Service use (session data, uptake, demographic data) |
| Beeber 2013 | To test interpersonal psychotherapy combined with parenting enhancement on depressive symptoms and parenting behaviour, compared with an equal attention-control condition. | Randomised trial. | 226 mothers of Early Head Start infants in North-Eastern and South-Eastern US states. | Low-income mothers. | Interpersonal therapy with parenting enhancement delivered in-home by psychiatric mental health advanced practice nurses  Setting - In-home, USA | Type – intervention  Strategies – PPS, OHBC | Attention-control delivered by generalist nurses. | - Clinical outcomes related to depression and parent-child interaction |
| Brown 2014 | To assess whether an IAPT service is delivering an equitable service in a London borough. | Cross-sectional analysis | 4,781 IAPT patients in Southwark (3,738 GP referred and 482 self-referred) and 196 SELCOH patients with mental health problems. | Access based on age, gender, ethnicity, occupational status and benefits status. | Increasing Access to Psychological Therapy (IAPT).  Setting – UK mental health services | Types – access, intervention  Strategy - IASCT | Patients in the South East London Community Health study (SELCOH). | - Access rates, service use patterns |
| Clarke 2014 | To evaluate the immediate and long-term impact of the 'Zippy's Friends' programme and to determine the impact of implementation fidelity on programme outcomes. | Cluster randomised controlled trial of Zippy's friends, with assessments before (Group 1), immediately after (Group 2) and 12-months post (Group 3). | 44 mixed-gender schools (766 children) with a designated disadvantaged status. | Socioeconomic disadvantage. | Zippy's Friends: a school-based emotional wellbeing programme.  Setting – schools in Ireland | Type – prevention  Strategies – DET, EC | Pre and post | - Emotional and behavioural outcomes, including emotional literacy |
| Clemans-Cope 2015 | To examine access to care and care experiences under the Children's Health Insurance Program (CHIP) compared to private coverage and being uninsured in 10 states. | Survey of CHIP enrollees in 10 states | Children in the CHIP program. | Socioeconomic disadvantage. | Children's Health Insurance Program.  Setting – health care settings in the USA | Type – access  Strategy - PFIP | Experiences under private coverage or in 10 uninsured in states. | - Parents ratings for confidence in meeting needs and affordability of care - Use of health services - Adequacy of access to health care - Unmet health needs |
| Comiskey 2015 | To evaluate the first implementation and impact of the World Health Organization (WHO) model among urban disadvantaged school children in Ireland from 2008 to 2012. | Mixed-methods: A process evaluation-mapped implementation and a three-year cohort study, along with focus groups, semi-structured interviews and documentary analysis. | Over 600 children in five intervention schools. | Socioeconomic disadvantage. | The World Health Organisation's ‘Healthy Schools’ model.  Setting – schools in Ireland | Type – prevention  Strategies – DET, EC | Two comparison schools | - Clinical outcomes (measures of childhood wellbeing and mental health) - Process evaluation – role/involvement of family and schools in implementation |
| Cooley-Strickland 2011 | To evaluate the efficacy of a school-based anxiety prevention program among urban children exposed to community violence. | Randomised controlled trial | 98 3rd-5th grade students, 92% of whom were African American. | Urban African American youth; socioeconomic disadvantage. | Modified FRIENDS program: cognitive-behavioural anxiety prevention.  Setting – schools in the USA | Types – early intervention, prevention  Strategies – PPS, EC, OCA | Wait-list control. | - Clinical outcomes - anxiety symptoms - Exposure to community violence - Educational achievement |
| Cupples 2011 | To examine the effect of an innovative tailored peer-mentoring programme, based on perceived needs, for first-time mothers in socio-economically deprived communities. | Randomised controlled trial | 343 first time mothers | Mothers; socioeconomic disadvantage. | Peer-mentoring by a lay-worker fortnightly during pregnancy and monthly for the following year (home visits and telephone contact).  Setting - home-based, Ireland. | Types – early intervention, prevention  Strategies – EC, DET | Wait-list control | - Infant psychomotor and mental development |
| Day 2012 | To evaluate the effectiveness of a peer-led parenting intervention delivered to socially disadvantaged families. | Randomised controlled trial | 116 index children aged 2-11 years. | Young people; socioeconomic disadvantage. | Manualised parenting programme delivered to groups of parents by trained peer facilitators from the local community.  Setting - schools and community-based, UK | Types – early intervention, intervention  Strategies – EC, PPS | Wait-list control. | - Child problems (number and severity) - parental stress, - parenting competencies |
| Dowrick 2013 | To increase equity of access to high-quality primary mental health care for underserved groups. | Mixed-methods: evidence review, model development, quasi-experimental model testing, then a feasibility/ acceptability test with qualitative elements. | 57 patients with unmet needs, identified by a GP. | Ethnic minorities; older people. | Improving Access to Mental Health in Primary Care (AMP): A culturally sensitive well-being intervention with individual, group and signposting elements.  Setting - primary care, UK. | Type – access  Strategies – EC, DET, OCA | No-intervention comparator | - Clinical outcomes (depression, anxiety) - Functioning (work and social adjustment measure) - Quality of life - Wellbeing - Service uptake and experience |
| Dowrick 2016 | To assess the effectiveness of the combined model in increasing access to and improving the quality of primary mental health care. | Multi-method: Quantitative and qualitative (focus groups) quasi-experimental design | 110 participants from four North-West UK localities (2 in Manchester & 2 in Liverpool). | Two location sites - older people; one location site - South Asian people; one location site - Somali people. | Improving Access to Mental Health in Primary Care (AMP).  Setting – primary care, UK | Type – access  Strategies – EC, DET, OCA | no-intervention comparators | - Service use (referral and uptake) |
| Dray 2017 | To evaluate the effectiveness of a universal, school-based intervention targeting resilience protective factors in reducing mental health problems in adolescents. | Cluster randomised controlled trial: school-based resilience intervention | 20 intervention and 12 control schools in socioeconomically deprived regions of New South Wales, Australia. | Young people; socioeconomic disadvantage. | A universal, school-based intervention targeting resilience protective factors.  Setting – schools in Australia | Types – early intervention, intervention  Strategies – EC, PPS | no intervention | - Mental health outcomes (strengths and difficulties questionnaire), internalising problems, and prosocial behaviour |
| Eames 2015 | To evaluate the eight-week Mindfulness-Based Wellbeing for Parents (MBW-P) programme. | Pre-/ post- single group comparison. | 23 socioeconomically disadvantaged mothers. | Socioeconomic disadvantage. | Mindfulness-based Wellbeing for Parents: an 8-week programme delivered in weekly, 2-hour sessions - aimed to emphasise wellbeing rather than reduction of stress.  Setting – NHS, UK | Types – early intervention, prevention   - Strategy- PPS | Pre/post | - Clinical outcomes - parent report of mental health: depression, rumination, stress, sense of mindfulness and wellbeing |
| Emery-Tiburcio 2017 | To determine whether the BRIGHTEN Program, an individually tailored, interdisciplinary "virtual" team intervention, would equally meet the needs of a highly diverse sample of older adults with depression. | Pre/ post single-group comparison. | 131 older adults (African-American, Hispanic, Non-Hispanic White). | Older adults; ethnicity. | An individually tailored, interdisciplinary "virtual" team intervention (BRIGHTEN)  Setting – primary care, USA | Types – access, intervention  Strategies – ELLS, OCA, OT, PPS, RSCT | Pre/post | - Clinical outcomes (depression, mental health functioning) |
| Galano 2017 | To test the efficacy of a program tailored specifically to meet the needs of Latinas who experience Intimate Partner Violence (IPV). | Trial: adapted empowerment programme. | 93 low-income, mostly immigrant Latina women. | Latina; low-income; immigrants; women exposed to partner violence. | A Spanish-language adaptation of the MOM’s Empowerment Program, a 10-week group treatment program for IPV-exposed women.  Setting – community-based, USA | Type – intervention  Strategies – ELLS, OCA, PPS | Wait-list control | - Violence exposure - PTSD symptoms |
| Gardner 2009 | To investigate moderators of change in an empirically supported family-centred intervention (the Family Check-Up) for problem behaviour in early childhood. | Randomised controlled trial: Family check-up intervention | 731 2 - 3 year olds from low-income families with early-onset problem behaviour. | Children; low-income families. | Family Check-Up intervention for problem behaviour in early childhood.  Setting – community services, USA | Type – intervention  Strategy – PPS | no-intervention control group | - Early childhood problem behaviour - Parental substance use - Maternal depression - Partner relationship quality - Cumulative risk index |
| Gardner 2010 | To examine mediators and moderators of change in conduct problems, in a multiagency randomized trial of the Incredible Years parenting program. | Randomised control trial | 153 pre-schoolers at risk for conduct problems. | The sample was analysed based on indicators of socioeconomic disadvantage. | Incredible Years BASIC Parent Program.  Setting – home and community-based, UK | Type – early intervention, intervention  Strategies – DET, PPS | Wait-list control. | - Parent reported measures of child problem behaviour - Parent and family characteristics including parental depression - Observation measures of parent-child interaction |
| Gilman 2013 | To determine whether economic disadvantage reduces the effectiveness of depression treatments received in primary care. | Differential-effects analyses of an RC. | 514 patients with major depression or clinically significant minor depression. | Analysis based on socioeconomic disadvantage. | Prevention of Suicide in Primary Care Elderly: Collaborative Trial (PROSPECT)  Setting – primary care, USA | Type – intervention  Strategies – DET, PPS, RSCT | Usual-care arms | - Depression symptoms and remission - Suicidal ideation |
| Gonzales-Ball 2019 | ^a^To determine the effectiveness of child-teacher relationship training for economically disadvantaged children exhibiting disruptive behaviour. | Randomised control trial | 23 Head Start teachers (and their aides), and 20 economically disadvantaged children. | Socioeconomic disadvantage. | Child–Teacher Relationship Training (CTRT): a play-based model  Setting – not reported (in abstract) | Type – early intervention, intervention  Strategies –  DET, PPS | Active control group | - Children’s levels of disruptive behaviour |
| Gopalan 2015 | To report on the 6-month follow-up outcomes of an effectiveness study testing a multiple family group (MFG) intervention for clinic-referred youth (aged 7–11) with disruptive behaviour disorders (DBDs). | Block comparison design | 225 clinic-referred youth (aged 7–11) with disruptive behaviour disorders (DBDs) and their socioeconomically disadvantaged families. | Youth; socioeconomic disadvantage; majority were BAME or Latino. | Multiple Family Group - a manualised intervention with 6-8 families, composed of the youth, adult caregiver(s) and sibling(s).  Setting – community-based mental health clinics, USA | Type – intervention  Strategies – PPS, PRF | Service-as-usual (n = 95) | - Parent-report measures of child behaviour, social skills, and impairment across functional domains |
| Grote 2015 | To evaluate whether ‘MOMCare’ is associated with improved quality of care and depressive outcomes compared to intensive public health Maternity Support Services (MSS-Plus). | Randomised multisite controlled trial | 168 depressed, pregnant women in receipt of Medicaid. | Maternity; socioeconomic disadvantage. | A culturally relevant, collaborative care intervention, providing a choice of brief interpersonal psychotherapy and/or antidepressants (MOMCare). | Type – access, intervention  Strategies – OCA, PPS, PRF, RSCT | MSS-Plus | - Depression and PTSD severity - Functional improvement - Depression response and remission - Quality of depression care. |
| Grote 2016 | ^a^To assess the impact of comorbid PTSD on pregnant, socioeconomically disadvantaged womens' response to the MOMCare intervention, providing Brief Interpersonal Psychotherapy and/or antidepressants. | Secondary analysis of a multisite randomized controlled trial with blinded outcome assessment | 65% of the sample of 164 met criteria for probable comorbid PTSD. | Maternity; socioeconomic disadvantage. | A culturally relevant, collaborative care intervention, providing a choice of brief interpersonal psychotherapy and/or antidepressants (MOMCare).  Setting – public health, USA | Type – access, intervention  Strategies – OCA, PPS, PRF, RSCT | Maternity Support Services. | - Depression and PTSD severity - Functional improvement - Depression response and remission - Quality of depression care. |
| Grote 2009 | To determine whether culturally relevant, enhanced brief interpersonal psychotherapy (IPT-B) confer greater advantages to low-income, pregnant women than enhanced usual care for depression in this population. | Randomised controlled trial | 53 non-treatment-seeking, pregnant African-American and white patients receiving prenatal services. | Maternity; socioeconomic disadvantage. | Enhanced IPT-B - engagement session, followed by 8 acute IPT-B sessions before birth and maintenance IPT up to six months post-partum.  Setting - obstetrics and gynaecology clinics, USA. | Type – intervention  Strategies – OCA, PPS | Enhanced usual care. | - Depression diagnoses and symptoms - Social functioning |
| Habib 2015 | To evaluate the efficacy of culturally adapted CBT for psychosis (CaCBTp) in Pakistan in a pilot project. | Randomised controlled trial | 42 in-patients with diagnosed schizophrenia. | Low-income. | Culturally adapted CBT for psychosis.  Setting - in-patient settings, Pakistan. | Type – intervention  Strategies – OCA, PPS | Treatment as usual | - Clinical outcome measures for schizophrenia and psychosis symptoms - A measure of insight at end of treatment |
| Handley 2017 | To investigate whether treating maternal depression via IPT may lead to more widespread positive adaptation for offspring and mothers. | Randomised controlled trial | 125 socioeconomically disadvantaged mother-child dyads; predominantly African-American. | Racially and ethnically diverse; socioeconomic disadvantage. | Interpersonal therapy.  Setting – clinics, USA | Type – intervention  Strategies – OHBC, PPS | Enhanced community standard control group | - Clinical outcomes – depression measure, diagnostic interview - Infant behaviour and attachment measures - Maternal efficacy (self-report measure of self-efficacy relating to parenting role) |
| Harris 2011 | To explore potential disparities in Better Access treatment using epidemiological data from the 2007 National Survey of Mental Health and Wellbeing. | Epidemiological population-level analysis, taken from the National Survey of Mental Health and Wellbeing. | Population-level analysis. | Socioeconomic disadvantage. | Better Access programme.  Setting - health care settings, Australia. | Type – access, intervention  Strategies – IATS, IASCT | n/a | - Disability - Clinical measures – diagnosis (ICD-10 disorder), severity of symptoms, psychological distress, chronic physical health comorbidity - Treatment need - Service use including services used and frequency |
| Held 2015 | To examine the effects of a 4-week-long self-administered self-compassion training on trauma-related guilt, compared with a stress inoculation | Pilot intervention comparison | 27 homeless male veterans who were living in transitional housing facilities. | Homelessness. | Self-compassion training (n=13) and stress inoculation.  Setting - transitional housing facility, USA. | Type – intervention  Strategy - PPS | Stress inoculation (n = 14). | - changes in self-compassion, trauma-related guilt, and PTSD severity |
| Hepworth 2015 | To examine the impact of the 2012 southern Queensland Centre of Excellence in Aboriginal and Torres Strait Islander Primary Health Care employment of a psychologist and a social worker to integrate mental health care into its primary health care services. | Mixed-methods: qualitative open-ended interviews and data collection of referrals. | Users of the Aboriginal and Torres Strait Islander primary care services. | Aboriginal communities. | Introducing a psychologist and a social worker into primary care.  Setting - primary care, Australia. | Type – access  Strategy - RSCT | n/a | - Service access and practice – referrals, social worker and psychologist involvement, changes in clinical practice - Service user feedback and experience |
| Humayun 2017 | ^a^To determine effectiveness of Functional Family Therapy for offending and antisocial behaviour in UK youth. | Randomised controlled trial | 111 adolescents and their families recruited through youth offending services and other crime prevention agencies. | Adolescents; youth offenders. | Functional Family Therapy + Management as Usual.  Setting -unclear, UK. | Type – intervention  Strategy - access | Management as usual. | - Parenting behaviour - Parent-child relationship - Offending and anti-social behaviour |
| Hutchings 2017 | To evaluate the effectiveness of the Incredible Years Toddler Parenting Programme with parents of 1‐year‐old and 2‐year‐old children recruited by staff in disadvantaged Flying Start areas across Wales. | Randomised controlled trial | 89 families with a child aged between 12 and 36 months at baseline. | Socioeconomic disadvantage. | Incredible Years Toddler Parent Programme.  Setting -  family centres, UK. | Type – early intervention, intervention  Strategy – DET, PPS | Wait list control | - parental mental health - parental competence - child behaviour and development - parent-child interaction |
| Jiminez 2015 | To compare the effect of problem-solving therapy against a health-promotion intervention (dietary practices) on health-related quality of life (HRQoL) and examine if there is a differential effect on non-Latino white patients and African American patients between the two interventions. | Secondary analysis of data from a randomized depression prevention trial | 247 older adults (154 non-Latino white, 90 African American, 3 Asian) | Ethnic minorities; older people. | Problem-Solving Therapy for Primary Care (PST-PC) vs coaching in dietary practices (DIET).  Setting - primary care, USA. | Type – intervention  Strategies – PPS, RSCT | Coaching in dietary practices (DIET). | - Health-related quality of life including mental health related quality of life. |
| Jongeneel-Grimen 2016 | To assess the mental health impact of an urban regeneration programme implemented from 2008 onwards in the 40 most deprived districts in the Netherlands. | Multi-level logistic regression models of cross-sectional data from the annual Health Interview Survey. | 1,445 adults in target districts and 44,795 adults across the rest of the Netherlands. | Deprived districts. | Urban regeneration program.  Setting – urban communities, Netherlands | Type – prevention  Strategies – EC, OCR | Comparable control districts | - Trends in perceived mental health related to intensity of the urban regeneration programme and sociodemographic confounders |
| Katon 2015 | To evaluate whether an obstetrics-gynaecology clinic-based collaborative depression care intervention is differentially effective compared with usual care for socially disadvantaged women with either no health insurance or with public coverage compared with those with commercial insurance. | Two-site randomised controlled trial, with an 18-month follow-up. Analysed based on insurance status | Women from mixed socioeconomic backgrounds (n = 136), and a county hospital serving an underserved, racially-diverse population (n = 69). | Women; socioeconomic disadvantage. | An obstetrics-gynaecology clinic-based collaborative depression care intervention.  Setting - clinic-based, USA. | Type – access  Strategy - RSCT | Usual care | - Depression outcomes – symptoms, severity and patient-rated improvement |
| Kenyon 2016 | To examine the effectiveness of lay support to improve maternal and child outcomes in disadvantaged families. | Prospective, pragmatic, individually randomised controlled trial | 1324 nulliparous women from disadvantaged families. | Maternity; socioeconomic disadvantage. | Lay support - Pregnancy Outreach Workers (POWs) were trained to provide individual support and case management for the women including home visiting from the point of randomisation to 6 weeks after birth.  Setting - 3 maternity trusts, UK | Type – access, intervention  Strategy - EC | Standard maternity care | - Antenatal visits attended - Postnatal Depression measure - maternal and neonatal birth outcomes - maternal self-efficacy, and mother-to-infant bonding - child development - breastfeeding - immunisation uptake |
| Kuosmanen 2017 | To examine the feasibility of delivering a computerized cognitive behavioural therapy gaming intervention (SPARX-R) for young people (age 15–20 years) who have left school early and are attending Youthreach, an alternative education program in Ireland. | Randomised controlled trial | 146 students in alternative education. | Young people; early school leavers - inferred socioeconomic risk. | A computerized cognitive behavioural therapy gaming intervention (SPARX-R) for young people who have left school early  Setting – youth reach centres, Ireland. | Types – early intervention, prevention  Strategies – OT, PPS | No-intervention control | - Clinical outcomes – measures of depression, anxiety, mental wellbeing, coping and emotional regulation |
| Legrand 2014 | To examine the possible mediating role of physical self-perceptions, physical self-esteem, and global self-esteem in the relationships between exercise and depression in a group of socioeconomically disadvantaged women with elevated symptoms of depression. | Randomised controlled trial | 44 female residents of a low-income housing complex. | Women; socioeconomic disadvantage. | 7-week exercise training group program.  Setting - unclear from abstract | Types – intervention  Strategy – DET | Wait-list group. | - Depression - Physical self-perceptions - Self-esteem |
| Leijten 2017 | To examine whether the Incredible Years parenting intervention can successfully reach and benefit families with socioeconomic disadvantaged and ethnic minority backgrounds in the Netherlands. | Randomised controlled trial | 154 families from a wide range of socioeconomic and ethnic backgrounds. Children were 3-8 years old and 65% of the children met DSM-IV criteria for oppositional defiant disorder, conduct disorder, and/or attention-deficit hyperactivity disorder. | Socioeconomic disadvantage; ethnic minorities. | BASIC Incredible Years parenting intervention.  Setting - child and adolescent outpatient psychiatry clinics and elementary schools serving deprived neighbourhoods, Netherlands. | Types – early intervention, intervention  Strategies – DET, PPS | Waiting list control. | - Child mental health and behaviours measures, including parent ratings of aggression and a diagnostic interview on child behaviour - Parent practices interview and parenting stress measure |
| Lovell 2014 | To develop a culturally sensitive wellbeing intervention with individual, group and sign-posting elements, and test its feasibility and acceptability for patients from ethnic minorities and older people. | Exploratory randomised controlled trial | 57 patients from 4 disadvantaged localities in the north-west of England. | Socioeconomic disadvantage; ethnic minorities; older people. | A culturally sensitive wellbeing intervention (comprising of a patient-centred assessment and a collaborative choice of pathway; individual or group intervention, or signposting.  Setting - primary care, UK | Types – access, early intervention  Strategies – OCA, PPS, RCST | Usual care. | - Mental health outcomes - global distress, depression and anxiety - Work and social adjustment - Health related quality of life |
| Ludwig 2012 | To examine the long-term effects of moving to less distressed neighbourhoods on broad measures of the well-being of low-income adults. | Data analysis of Moving to Opportunity (MTO), a randomised housing experiment. | 4,604 low-income public housing families living in high-poverty neighbourhoods, who enrolled in the MTO between 1994-1998. | Socioeconomic disadvantage. | Moving to Opportunity: via random lottery, MTO offered some public housing families but not others the chance to move into a less-distressed area.  Setting - high-poverty neighbourhoods, USA. | Type – prevention  Strategy – O-NOS | - Traditional voucher group - Control group | - Census-level address information - Summary indices of long-term adult outcomes in economic self-sufficiency, physical health and mental health - Self-reported subjective well-being |
| Lwembe 2017 | To evaluate a pilot cross-sector initiative - bringing together public health, a community group, primary mental health teams and patients - in using co-production approaches to deliver a mental health service to meet the needs of the black and minority ethnic communities. | Qualitative research methods, including semi-structured interviews and focus groups. | 25 service users of a community mental health service amongst the black and minority ethnic community. | BAME. | Co-production of a mental health service.  Setting - community mental health service, UK. | Type – access  Strategy - EC | No comparator | - Retention rates |
| Mathias 2018 | To assess the effectiveness of a brief mental health and resilience intervention among disadvantaged young women in urban North India. | Uncontrolled repeated measures design | 106 young women residing in a slum in Dehradun, Uttarakhand. | Socioeconomic disadvantage; young women. | A 15-module mental health and resilience curriculum  Setting - slums, India | Type – intervention  Strategies – DET, EC | n/a | - Self-efficacy - Resilience - Anxiety - Depression - Gender attitudes |
| McEvoy 2017 | To reflect upon the Eis Leader initiative which sought to improve uptake of NHS mental health services for the Orthodox Jewish community. | Mixed methods study: qualitative and quantitative data. | Data regarding the Haredi Jewish community, and 12 informants in Salford, Greater Manchester (inc. Rabbis, service-user, PWP's, leaders of Jewish community organisations, a practice manager…) | Minority ethnic community: Orthodox Jewish Community. | Eis Ledader ('time to talk') project to address low uptake of NHS MH services with Jewish Community. | Type – intervention  Strategies – EC, IASCT, OCA | n/a | - 6 levels of analysis – who was involved, the language, concepts and logic used, perceptions about relationships, contextual factors on barriers and facilitator, salient concerns, outcomes of processes over time. |
| McGilloway 2012 | To test the effectiveness of the Incredible Years BASIC parent training program for children with behavioural problems in urban areas characterized by high levels of disadvantage. | Randomised controlled trial | 149 parents with a child aged between 32-88 months and scoring above a clinical cut-off on the Eyberg Child Behaviour Inventory. | Socioeconomic disadvantage. | Incredible Years BASIC parent training program for children with behavioural problems. | Types – early intervention, intervention  Strategies – DET, PPS | Waiting-list control | - Child behaviour - Parenting skills - Parent well-being   (parent reported and observational) |
| McGilloway 2014 | To examine the effectiveness of the Incredible Years Basic parent programme in reducing child conduct problems and improving parent competencies and mental health. | Repeated measures pre-/ post- intervention | 103 families and their children (aged 32–88 months), who previously participated in a randomised controlled trial of the Incredible Years Basic parent programme. | Socioeconomic disadvantage. | Incredible Years BASIC parent training program for children with behavioural problems.  Setting - community-based, Ireland | Types – early intervention, intervention  Strategies – DET, PPS | Pre/post baseline to (12-month follow-up) | - Child conduct problems - Parenting behaviour - Parental mental health - Service use - Associated costs |
| Mehdipanah 2014 | To evaluate the effects of the Neighbourhoods Law on the health and health inequalities of residents in the intervened neighbourhoods in the city of Barcelona. | Quasi-experimental pre-design and post- design | Adult residents in five intervened neighbourhoods and eight non-intervened neighbourhoods. | Socioeconomic disadvantage. | Urban renewal of 5 neighbourhoods  Setting - low-SES neighbourhoods, Spain | Type – prevention  Strategies – EC, OCR | 8 non-intervened neighbourhoods with similar SES characteristics. | - Self-rated health and mental health in 2001, 2006 and 2011. |
| Meir 2014 | To construct and examine the effectiveness of a specialized group intervention program to enhance children’s self-efficacy and mental health. | Repeated measures design: pre-/post- assessments | 70 children aged 8–12 of illegal migrant workers in Israel. | Youth; children of immigrant workers. | Group intervention program to enhance children’s self-efficacy and mental health.  Setting - unclear, Israel | Types – early intervention, intervention  Strategies – OCA, PPS | Control (not clear what the control is). | - self-efficacy, emotional, behavioural and social difficulties child-report and teacher-report measures |
| Mendelson 2015 | To explore whether the RAP Club program was associated with positive outcomes for students with varying baseline depression levels to evaluate its potential as a classroom-wide intervention. | Randomised controlled pilot study | 49 seventh and eighth graders at two urban public schools serving low-income communities | Socioeconomic disadvantage. | RAP Club, a 12-session school-based trauma-informed group intervention co-facilitated by a mental health counsellor and young adult community member that utilizes evidence-based cognitive-behavioural and mindfulness strategies.  Setting - school-based, USA | Types – early intervention, intervention  Strategies – DET, PPS, RSCT | Regular school programming | - Teacher reported and student reported measure of student mental health, emotional regulation, behaviour and coping skills, educational outcomes, and social competence |
| Meyers 2014 | To determine whether depression screening and treatment improved depression scores of a medically uninsured, mostly African American primary care population. | Prospective repeated-measures design: baseline vs 8,12, and 14 weeks. Treatment arms: (1) usual care; (2) usual care and psychotherapy; (3) usual care and education and psychotherapy; and (4) usual care and education. | 674 uninsured patients, predominantly primary care African-American population. | Socioeconomic disadvantage. | Depression screening and treatment in a medically uninsured population.  Setting – primary care, USA. | Types - access, intervention  Strategies – DET, IATS, PPS | Usual care | - Depression prevalence and scores |
| Michelson 2014 | To develop and test the feasibility of a peer-led parenting intervention for parents of adolescent children. | Formative evaluation using a mixed-method cohort design. | 41 parents seeking help with managing behavioural difficulties of an index adolescent child (aged 11-17 years). | Socioeconomic disadvantage; predominantly minority ethnic communities. | A structured, group-based intervention ('Living with Teenagers') delivered by trained peer facilitators.  Setting - Socially deprived community sites in London, UK. | Types – access, intervention  Strategies – DET, EC | n/a | - Uptake and completion rates - Social validity (satisfaction measures) - parent-reported adolescent behaviour and mental health, - parenting satisfaction, expressed emotion and disciplinary practices |
| Miech 2008 | To examine the potential for the ‘Comprehensive Community Mental Health Services for Children and Their Families’ program to address health disparities. Specifically, to examine whether program sites disproportionately provide services within their catchment areas for youth who come from poor families, who are Black, and who are Hispanic. | Meta-analysis of data from study sites compared with comparison sites. | 19,189 youth (from 45 sites) who were enrolled in program sites from 1997-2005, with a diagnosed DSM-IV condition. | Youth; socioeconomic disadvantage; BAME. | ‘Comprehensive Community Mental Health Services for Children and Their Families’ program  Setting - community settings, USA. | Types – access intervention  Strategies – EC, IASCT, OCA, RSCT | A comparison catchment area. | - Analysis examined the proportion of children enrolled in the programme with particular characteristics including race and ethnicity and household income |
| Pachankis 2015 | To test the preliminary efficacy of a transdiagnostic cognitive behavioural treatment adapted to improve depression, anxiety, and co-occurring health risks (i.e., alcohol use, sexual compulsivity, condomless sex) among young adult gay and bisexual men. | Randomised controlled trial | 63 young gay and bisexual men. | Minority sexual orientation; LGBTQ. | ESTEEM (Effective Skills to Empower Effective Men) is a 10-session intervention based on the Unified Protocol for the Transdiagnostic Treatment of Emotional Disorders an individually-delivered cognitive behavioural treatment with efficacy for reducing stress-sensitive mental health disorders (e.g., depression, anxiety).  Setting - research office, USA. | Types –intervention  Strategies – OCA, PPS | Three-month waiting list. | - Alcohol Use Disorders Identification - Depression and anxiety measures - Sexual behaviour measure - Wellbeing and behaviour measures associated with sexual orientation - Social support |
| Pan 2011 | To provide a 6-month follow-up of a randomized pilot evaluation of standard one-session treatment (OST-S) versus culturally-adapted OST (OST-CA) with phobic Asian Americans. | Randomised pilot study | 30 phobic Americans of East Asian ethnicity - 90% had a primary fear of spiders. | Minority ethnicity. | OST-S is a brief in vivo exposure treatment. OST-CA is identical to OST-S except for the inclusion of seven research-based, cultural adaptations.  Setting - ‘In vivo’ (treatment settings), USA. | Type - intervention  Strategies –PPS, OCA | Standard one-session treatment  group and a self-help group | - Measures of distress - DSM symptom count from ADIS-IV |
| Pancer 2013 | To examine the long‐term impacts of Better Beginnings on the parents, families and communities of the three older cohort sites. | A quasi‐experimental longitudinal comparison site design | 959 families, 601 from the three Better Beginnings communities and 358 from the matched comparison communities. Data were gathered from only one child per family (born in 1989 or 1990) and one parent per family. | Socioeconomic disadvantage. | Better Beginnings, Better Future: designed to reduce children's problems, promote healthy child development, and enhance family and community environments in 3 three low SES communities. in the province of Ontario, Canada.  Setting – community-based, Canada | Type - prevention  Strategies – EC, OCR | Children and their families in two comparison neighbourhoods (with socio‐demographic similarity) | - Parents' health status and health risk behaviours (smoking/alcohol) - Parents' social and emotional functioning and   involvement with the Criminal Justice System   - Family stress and functioning - Community involvement and perceptions |
| Pearce 2016 | To compare the effectiveness of religious cognitive-behavioural therapy (RCBT) versus conventional CBT (CCBT) on reducing spiritual struggles in persons with major depressive disorder (MDD) and medical illness. | Randomised controlled trial | 132 enrolled - predominantly Christian (also Jewish, Muslim, Hindu or Buddhist). | Religious groups. | 50-minute sessions of either CCBT or RCBT administered over 12 weeks. Because participants had chronic medical illness, treatment sessions were conducted remotely by telephone (94%), Skype (5%), or instant messaging (1%).  Setting – remote (telephone, Skype), USA. | Type - intervention  Strategies – OCA, PPS | Conventional CBT | - Spiritual struggles - negative religious coping - Religiosity - Depression |
| Peng 2015 | To perform a psychological intervention on the mental health of left-behind middle school students and college students in rural areas by prescribing physical exercise. | Randomised controlled trial | Left-behind children from two classes in Grades 1 and 2 of two middle schools in a rural area. | Youth; 'Left-behind' students; rural area. | Physical exercise (12-week duration, n=62).  Setting - school-based, China. | Types – early intervention, prevention  Strategy– DET | Control group (n=59). | - Measures of obsession, intolerance, hostility, intense interpersonal relationship, sensitivity, anxiety, emotional imbalance and psychological imbalance |
| Pourmohamadreza-Tajrishi 2013 | To determine the effectiveness of emotional intelligence training on the mental health of deaf students. | Semi-experimental study with pretest and posttest design | 40 randomly selected male deaf students | Youth; physical impairment. | 12 sessions (each session lasts for 50 minutes; twice a week) of an emotional intelligence program  Setting - school-based; Iran. | Types – early intervention, prevention  Strategies – DET, EC | No intervention | - General Health Questionnaire (somatic symptoms, anxiety, social dysfunction and depression) |
| Renner 2011 | To examine the effectiveness of Culture-Sensitive and Resource Oriented Peer (CROP)—Groups for Chechen asylum seekers and refugees towards reducing post-traumatic symptoms, anxiety, and depression. | Randomised controlled pilot study | 94 refugees and asylum seekers from Chechnya. | Asylum seekers and refugees. | 15 sessions of Culture-Sensitive and Resource Oriented Peer (CROP) - Groups (also referred to in the abstract as CBT). Or 3 single sessions of EMDR.  Setting - unclear, Austria, Russia | Type –intervention  Strategies –EC, OCA, PPS | Eye-Movement Desensitisation and Reprocessing (EMDR) and wait-list controls | - Clinical outcomes -PTSD, anxiety and depression symptoms |
| Rhodes 2016 | Determine whether the Chronic Care Initiative (CCI) impacted the utilization and costs for Medicaid patients with chronic medical conditions and comorbid psychiatric or substance use disorders. | Analysis of Medicaid claims using difference-in-difference regression analyses to compare changes in utilization and costs for patients treated at CCI practices | 11,105 comorbid Medicaid patients treated in CCI practices and an equal number of propensity-matched comparison patients treated in non-CCI practices. | Socioeconomic disadvantage; co-morbid physical health problems. | Chronic Care Initiative: a large state-wide patient-centred medical home (PCMH) initiative in Pennsylvania in place from 2008-2011.  Setting - CCI and non-CCI practices; USA. | Type – access  Strategies – IASCT, IATS | Propensity score-matched patients treated at comparison non-CCI practices. | - Changes in total per-patient costs from 1 year prior to 1 year following an index episode period. - Utilization - Costs for emergency department, inpatient and outpatient services |
| Roberts 2010 | To investigate the efficacy of the Aussie Optimism Program. | Randomised controlled trial | 496 Grade 7 students, aged between 11 and 13 years, from 12 government primary schools in Perth - randomly sampled from the lowest decile of SES. | Socioeconomic disadvantage. | Aussie Optimism Program: two components - Social Life Skills and Optimistic Thinking Skills  Setting - school-based, Australia | Types – early intervention, prevention  Strategy - DET | Usual health education lessons. | - Child depression and anxiety measures - Parent reported child behaviour - Child attributional style measure (examines ideas around causality - Social skills measure |
| Sandner 2018 | To evaluate the effects of home visiting targeted towards disadvantaged first-time mothers on maternal and child health outcomes. | Data analysis of a randomised controlled trial with longitudinal survey data and administrative health data. | 755 expectant mothers | Maternity; socioeconomic disadvantage. | Pro Kind program: an adaptation of the U.S. Nurse Family Partnership - provides instructions for home-visit frequency, employee selection, teaching material and guidebooks.  Setting - Home-based, Germany | Types – early intervention, prevention  Strategy - OHBC | Control group | - Health care utilisation - Diagnoses and prescriptions - Interview responses on maternal health behaviour and maternal and physical health. |
| Seabra-Santos 2018 | To analyze the impact of an evidence-based teacher-training program, the Incredible Years Teacher Classroom Management (IY-TCM), on the behavior of economically disadvantaged Portuguese children. | Experimental randomized controlled between-group design, with pre- and post-intervention assessments. | 538 children between the ages of 3 and 6. Schools were selected for their high percentage of children from economically disadvantaged backgrounds. | Youth; socioeconomic disadvantage. | IY-TCM program, implemented in six monthly workshops, and four sessions of individual in-class support.  Setting - school-based, Portugal | Types – early intervention, intervention  Strategies – DET, EC, PPS | Control group of 492 children equivalent with regard to gender and age. Schools were matched based on economic need and classroom size. | - Kindergarten behaviour scale |
| Shaikh 2016 | To evaluate the outcomes of the Global Fund-supported Pehchan programme. | Pre- and post-intervention cross-sectional survey design with retrospective analysis of programmatic data. | 268 transgender participants in six Indian states from a total of 48,280 transgender people served by Pehchan through 186 community-based organizations. | Transgender communities. | Global Fund-supported Pehchan programme: aimed to strengthen community systems and provide HIV, health, legal and social services to transgender communities through a rights-based empowerment approach.  Setting - community-based, India | Types – access, intervention, prevention  Strategies - DET, EC, OCR, PPS | pre/post | - Access to health care, social care and legal advice - Self-efficacy |
| Shebeer 2017 | To evaluate an Internet-facilitated cognitive-behavioural treatment intervention for depression, tailored to economically disadvantaged mothers of young children. | Randomised controlled trial | 266 Economically disadvantaged mothers of preschool aged children, who reported elevated levels of depressive symptoms. | Mothers; socioeconomic disadvantage. | Mom-Net: 8-sessions of internet-facilitated CBT.  Setting - remote, USA | Types – access, intervention,  Strategies – OCA, OT, PPS | Motivational interviewing + Referral to Services (MIRS). | - Depression measures - Outcome from diagnostic clinical interview |
| Sheeber 2012 | To develop and pilot an Internet-facilitated cognitive-behavioural treatment intervention for depression, tailored to economically disadvantaged mothers of young children. | Randomised controlled pilot | 70 mothers (of children enrolled in Head Start), who reported elevated levels of depressive symptoms. | Mothers; socioeconomic disadvantage. | Mom-Net: 8-sessions of internet-facilitated CBT.  Setting - remote, USA | Types – access, intervention,  Strategies – OCA, OT, PPS | Delayed intervention/ treatment as usual. | - Depression measures |
| Snowden 2016 | To investigate whether a new funding opportunity to finance mental health treatment, provided to autonomous county-level mental health systems without customary cost sharing requirements, equalized African American and White children's outpatient and emergency treatment expenditure inequalities. | Controlled cross-sectional and longitudinal analysis of Medicaid claims for mental health services delivered to youth (under 18) from July 1 1991 to June 30 2007. | Medicaid claims over 64 quarters (July 1991 to June 2007). | Youth; ethnicity; socioeconomic status. | Settlement-mandated increased Early Periodic Screening, Diagnosis and Treatment (EPSDT) treatment funding. | Type – access  Strategy - PFIP | n/a | - Expenditures on outpatient care and emergency services |
| Snowden 2017 | To investigate whether total Latino-White expenditure disparities declined when autonomous, county-level mental health plans receive funds free of customary cost-sharing charges, especially when they capitalized on cultural and language-sensitive mental health treatment programs as vehicles to receive and spend treatment funds. | Segmented regression for interrupted time series on county level treatment systems observed over 64 quarters. | Medi-Cal paid claims for per-user total expenditures for mental health services delivered to children and youth (under 18 years of age) during a study period covering July 1, 1991 through June 30, 2007. | Socioeconomic disadvantage; socioeconomic status. | Settlement-mandated increased EPSDT treatment funding.  Setting - outpatient and emergency services, USA | Type – access  Strategy - PFIP | n/a | - Medi-Cal paid claims for per-user total expenditures for mental health services |
| Steinberg 2014 | To evaluate the effect of a weight gain prevention intervention (Shape Program) on depression among socioeconomically disadvantaged overweight and obese women from Black communities. | Analysis of a randomized controlled trial | 5 central North Carolina community health centres. | BAME; socioeconomic disadvantage. | Shape Program: 12-month electronic health-based weight gain prevention intervention with tailored behaviour change goals, self-monitoring of goals, tailored skills training materials, individual counselling calls with a dietician and a year-long YMCA membership  Setting - community-based, USA. | Types – early intervention, prevention  Strategy - DET | Usual primary care | - Depression measure |
| Stevens 2018 | To examine the effectiveness of a coordinated perinatal mental health care model, focusing on socially-disadvantaged, ethno-racial minority women, with an intersectional-feminist perspective. | Effectiveness study to test generalisability of an efficacious intervention to actual clinical practice. | 67 perinatal women (64% African American or Hispanic/Latina) referred by medical providers at an urban teaching hospital. | Perinatal; BAME. | A coordinated perinatal mental health care model developed based on intersectionality theory.  Setting - outpatient, USA | Type –intervention  Strategies – OCA, PPS, RSCT | n/a | - Treatment engagement - Depression, anxiety and PTSD measures |
| Stewart 2017 | To conduct a proof-of-concept pilot study of Trauma-Focused-CBT (TF-CBT) delivered to underserved trauma-exposed youth and their nonoffending caregivers via telehealth technology (i.e., via one-on-one videoconferencing) either at their schools or their homes. | Proof-of-concept pilot | 15 underserved trauma-exposed youth (e.g. history of sexual or physical abuse, loss of a loved one, witness to crime, or multiple traumas). | Youth; ‘underserved’ (e.g. rural or remote living; low SES, BAME). | TF-CBT delivered via telehealth technology (i.e. via one-on-one video-conferencing), aimed at addressing barriers in access to TF treatment.  Setting – remote, USA | Types –access, intervention  Strategies – OT, PPS | n/a | - Delivery strategies - Technical performance of equipment - Safety issues - Service use - number of sessions attended and completion rates - Clinical outcomes - self- and parent- reported |
| Sullivan 2013 | To examine the effects of a collaborative care intervention for anxiety disorders in primary care on lower income participants relative to those with higher incomes. | Analysis of a randomised trial | 1004 primary care patients with panic disorder, social anxiety disorder, generalized anxiety disorder or posttraumatic stress disorder. | Analysis based on socioeconomic disadvantage. | Coordinated Anxiety Learning and Management (CALM): a flexible, collaborative care delivery model for primary care anxiety treatment.  Setting - primary care, USA. | Types –access, intervention  Strategies – OCA, PPS, RSCT | Usual care | - Self-report measures of mental health - Health rated quality of life - Income |
| Ter Heide 2011 | To examine feasibility of participation in a randomised trial for this complex population and to examine acceptability and preliminary efficacy of EMDR. | Feasibility study in preparation for an RCT | 20 traumatised refugees or asylum seekers. | Asylum seekers and refugees. | 11 sessions of EMDR.  Setting – specialist psycho-trauma treatment centre, the Netherlands. | Type – intervention  Strategy – PPS | Stabilisation | - Symptoms of PTSD, depression and anxiety - Health related quality of life |
| Tighe 2017 | To evaluate the effectiveness of a self-help mobile app (ibobbly) targeting suicidal ideation, depression, psychological distress and impulsivity among Indigenous youth in remote Australia. | Randomised pilot study | 61 Indigenous Australians aged 18–35 years. | Remote regions; indigenous communities. | An app (ibobbly) which delivered acceptance-based therapy over 6 weeks  Setting - remote and very remote communities, Australia. | Types –access, intervention  Strategies – DET, EC, OCA, OT, PPS | Wait list / delayed treatment (wait listed for 6 weeks before receiving intervention) | - Depressive symptoms - Suicidality |
| van der Gucht 2015 | To investigate the efficacy of a Mindfulness-Based Intervention (MBI) for economically disadvantaged people. | Two-baseline, post- and follow-up within-subjects design. | 42 low-income people. | Socioeconomic disadvantage; receiving social welfare benefits. | The MBI program adhered to a standardized protocol developed from the MBSR (Mindfulness-Based Stress Reduction) and MBCT (Mindfulness-Based Cognitive Therapy) manuals.  Setting welfare centres, Belgium. | Type –intervention  Strategies – OCA, PPS | Within subjects | - Depression, anxiety and stress outcomes - Cognitive vulnerability processes - Development of mindfulness skills. |
| van der Waerden 2013 | To evaluate the effects of the Exercise Without Worries (EWW) multidisciplinary preventive intervention aimed at reducing stress and depressive symptoms among low-SES women. | Randomised controlled trial | 161 Dutch low-SES women (20–55 years) with depressive symptoms or who suffered from stress-related complaints. | Socioeconomic disadvantage. | EWW - exercise/ psycho-education intervention integrating group-based exercise with cognitive-behavioural techniques.  Setting - unclear, the Netherlands. | Types –access, prevention  Strategies – DET, OCA,  PFIP | Exercise only and waiting list control (WLC) | - Depressive symptoms - Perceived Stress - Dutch Recent Life Events Questionnaire |
| Velasquez 2015 | To evaluate the impact of participating in extracurricular Yoga workshops on the prevention of anxiety, depression, and aggression. | Randomised controlled trial | 296 students from a public school (operated by a private educational institution) in Bogotá, from Grades 5, 8, and 9. | Youth; socioeconomic disadvantage. | Yoga: Twenty-four 2-hr sessions were held for each of the five groups. An instructor, who was a school outsider and who was experienced in the conduction of Yoga trainings, led the sessions.  Setting – school-based, Colombia | Type – prevention  Strategy – DET | no participation | - Depression and anxiety outcomes - Peer assessment of aggressive behaviour - National test of Citizenship Competences |
| Walthery 2015 | To investigate the impact of the regeneration initiative, New Deal for Communities (NDC), which ran from 1999 to 2011, on socioeconomic inequalities in health trajectories. | Latent Growth Curve modelling of residents in areas in which the NDC was implemented | 39 disadvantaged areas of England in which the NDC was implemented, compared with residents of comparator, non-intervention areas. | Socioeconomic disadvantage. | NDC: a regeneration initiative to tackle spatial inequalities in concentrated deprivation and health.  Setting - disadvantaged regions, UK. | Types – access, prevention  Strategies - EC, IASCT, OCR | Non-intervention areas | - Self-rated health and mental health - Self-rated life satisfaction |
| Webster-Stratton 2008 | To evaluate the Incredible Years (IY) Teacher Classroom Management and Child Social and Emotion curriculum (Dinosaur School) as a universal prevention program for children enrolled in Head Start, kindergarten, or first grade classrooms in schools selected because of high rates of poverty. | Randomised trial | Matched pairs of school – 153 teachers and 1,768 students. | Youth; socioeconomic disadvantage. | Incredible Years (IY) Teacher Classroom Management and Child Social and Emotion curriculum (Dinosaur School).  Setting – school-based, USA. | Types – early intervention, intervention  Strategies – DET, EC, PPS | A control condition (usual school curriculum) | - Observations of teacher classroom management and style - Observation of child conduct problems, emotional self-regulation and competence |
| Weisman de Mamani 2014 | To test whether a culturally informed treatment for schizophrenia (CIT-S) would out-perform psychoeducation (PSY-ED) in reducing post-treatment symptom severity (controlling for baseline symptoms) on the Brief Psychiatric Rating Scale. | Randomised controlled trial | 69 participants with schizophrenia; predominantly Hispanic/ Latino, Black or another minority White community. | BAME communities. | 15 sessions of CIT-S - a family focused, culturally informed treatment for schizophrenia patients and their caregivers: 15 sessions.  Setting -community settings, USA. | Type –intervention  Strategies – OCA, PPS | 3-session PSY-ED control condition | - Clinical outcome – symptom severity - Consumer satisfaction |
| White 2017 | To examine the association between neighbourhood regeneration and mental health. | Propensity Score Matching: Data linkage of information on regeneration activities mental health data from a cohort study before (2001) and after (2008) regeneration. | 4,197 participants in intervention areas and 6,695 in control areas. | Socioeconomic disadvantage. | ‘Communities First’: an areawide socioeconomic regeneration program implemented in deprived neighbourhoods in Wales.  Setting - deprived neighbourhoods, UK. | Type – prevention  Strategies – EC, OCR | 75 control areas | - Self-reported mental health outcomes - Socioeconomic factors |
| Williams 2015 | To develop a cognitive therapy program to reduce mental distress among hearing-impaired employees. | Controlled pilot study | 15 people with hearing impairment (mild to moderate). | Sensory impairment. | A weekly two-hour CBT course over eight weeks (16 hours in total): presented in Norwegian, with an inductive hearing loop system.  Setting – therapy sessions, Norway. | Type – intervention  Strategies – ELLS, OCA, PPS | Treatment as usual (traditional audiological rehabilitation program) | - Anxiety and depression measure - Communication – Conversation Tactics Checklist |
| Yahyaee 2015 | To determine the effect of attribution retraining group program on depression of students with learning disabilities in city of Tehran. | Quasi-experimental: pre-/post- assessments | 36 students (27 males, 9 females) from centers for learning disorders | Learning disability. | Eleven group sessions of attribution retraining program twice a week. Each session lasts for 45 minutes.  Setting – Center for Learning Disabilities, Iran. | Type – intervention  Strategy – DET | Control (not clear what the control is) | - Children’s depression inventory |
| Zandkarimi 2018 | To evaluate the effect of group training with non-violence communication (NVC) method on stress, anxiety and depression of young girls. | Randomised controlled | 50 participants from the charity centers of Tehran suburbs, aged from 11- 18, the vulnerable socio-economic income and the stress cut off = 12. | Youth; socioeconomic disadvantage. | Eight group training sessions of NVC.  Setting - charity centres, Iran. | Type – early intervention  Strategy - DET | Control (no intervention) | - Depression, anxiety and stress scale |

[TABLE 1 LEGEND]

^a^ Study aims were not explicitly defined by the study authors and have been inferred in some cases.

AMP = access to mental Health in primary care; ATAPS = Australian Access to Allied Psychological Services; BAME = Black, Asian and Minority Ethnic; CaCBTp = culturally adapted cognitive-behavioural therapy for psychosis; CALM = Coordinated Anxiety Learning and Management; CBT = cognitive-behavioural therapy; cCBT = computerised CBT; CCBT = conventional CBT; CCI = Chronic Care Initiative; CHIP = Children’s Health Insurance program; CIT-S = culturally informed treatment for schizophrenia; CROP = Culture-Sensitive and Resource Oriented Peer; CTRT = child- teacher relationship training; DBD = disruptive behaviour disorder; DET = Delivering education and training; DSM-IV = Diagnostic and Statistical Manual of Mental Disorders, 4^th^ Edition; EC = Engaging the community; ELLS = Enhancing language/literacy and communication; EMDR = Eye-Movement Desensitisation and Reprocessing; EPSDT = Early Periodic Screening, Diagnosis and Treatment; ESTEEM = Effective Skills to Empower Effective Men; EWW = Exercise Without Worries; GP = general practitioner; HRQoL = Health-Related Quality of Life; IAPT = Improving Access to Psychological Treatments; IASCT = Improving access to support care and treatment for mental health problems; IATS = Improving access to testing and screening; ICD-10 = International Classification of Diseases, 10^th^ Edition; IPT = interpersonal therapy/treatment; IPT-B = brief interpersonal therapy/treatment; IPV = Intimate Partner Violence; IY = Incredible Years; IY-TCM = Incredible Years Teacher Classroom Management; LGBTQ = Lesbian, gay, bisexual, transgender, queer; MBCT = mindfulness-based cognitive therapy; MBI = mindfulness-based intervention; MBSR = mindfulness-based stress reduction; MBW-P = mindfulness-based wellbeing for parents; MDD = major depressive disorder; MFG = multiple family group intervention; MIRS = Motivational interviewing + Referral to Services; MSS = maternity support services; MTO = moving to opportunity; NDC = New Deal for Communities; NHS = National Health Service; NVC = non-violence communication; OCA = Other – culturally adapted interventions; OCR = Other – community revitalisation; OHBC = Other – home-based care; O-NOS= other – not otherwise specified; OST-CA.= culturally-adapted one-session treatment; OST-S = one-session treatment; OT = Other – technology; PCMH; patient-centred medical home; PFIP = Providing financial incentives or removing financial barriers; POW = pregnancy outreach worker; PPS = Providing psychological support; PRF = Providing reminders and feedback; PSY-ED = psychoeducation; PST-PC = Problem Solving Therapy for Primary Care; PTSD = post-traumatic stress disorder; RCT = randomised controlled trial; RCBT = religious cognitive-behavioural therapy; RSCT = Restructuring the care team; SELCOH – South-East London community health study; SES = socioeconomic status; TF-CBT = Trauma-focused cognitive-behavioural therapy; YMCA = Young Men’s Christian Association; WHO = World Health Organization; WLC = waiting list control

Table 2: Research question 2 primary studies

| **Study ID (first author + year)** | **Study aim(s)** | **Study design** | **Population sample** | **Population characteristic/s associated with inequality** | **Intervention detail** | **Comparator** | **Outcomes** | **Currency** |
| --- | --- | --- | --- | --- | --- | --- | --- | --- |
| Gardner 2017 | To examine the benefits of the Incredible Years parenting programme in socioeconomically disadvantaged families including any differential effects for ethnic minority families and to explore the costs, cost effectiveness and long-term savings. | Individual participant data meta-analysis of randomised control trials. | 1799 families with children aged 2 to 10 years with ODD and CD Economic data were available for five UK and Ireland trials (maximum n=608). | CYP with ODD and CD from low SES and ethnic minority families. | The Incredible Years parenting programme.  Setting - Europe (UK, Netherlands, Ireland, Norway, Sweden and Portugal). | Waiting list, minimal intervention or care as usual | - Financial outcomes – cost, cost effectiveness, long term savings - Clinical outcomes – child behaviour | Not reported |
| O'Neill 2013 | To examine the cost-effectiveness of the Incredible Years parenting programme in reducing childhood conduct problems in Ireland. | Economic component (incremental cost effectiveness analysis, long-term cost benefit analysis) of a randomised controlled trial. | 132 families of children with conduct problems from low SES families. | CYP with oppositional defiant disorder and conduct disorder from low SES families. | The Incredible Years parenting programme.  Setting - Community-based services, Ireland (4 low socio-economic status urban areas). | Control group waiting list | - Service and financial outcomes – service usage and recurrent costs, cost effectiveness, cost-benefit - Clinical outcomes – child behaviour | Euros |
| Grote 2017 | To estimate the incremental benefit, cost and net benefit of MOMCare in women with major depression or PTSD including women from low SES and racially and ethnically diverse groups. | Randomised controlled trial. | 164 pregnant women with a probable diagnosis of depression or depression and PTSD who qualified for public health care. | Low SES ethnic minority mothers | MOMCare collaborative care depression intervention (choice of brief interpersonal psychotherapy or pharmacotherapy or both).  Setting - Public health system, USA. | Maternity Support Services. | - Depression and PTSD symptoms and estimated gain in symptom free days - Care cost - Incremental net benefit | US Dollars |
| McGilloway 2014 | To examine if the positive outcomes of an Incredible years group programme were maintained at 12 month follow up of an RCT and whether the use of health, social and special educational services would decrease in the longer run. | Randomised controlled trial and cost analysis. | 103 families of children aged between 32 and 88 months. | Low SES families | Incredible Years Basic parent programme.  Setting - Ireland | Pre/post baseline to (12-month follow-up) | - Service utilisation and cost - Child behaviour outcomes   Parental wellbeing and psychosocial functioning | Euros |
| Rhodes 2016 | To determine whether the Chronic care initiative (CCI) impacted utilization and costs for continuously enrolled Medicaid patients with chronic medical conditions and comorbid psychiatric or substance use disorders, a population who may disproportionally benefit from structural benefits of care coordination. | Analysis of Medicaid claims using difference-in-difference regression analyses to compare changes in utilization and costs | Continuously enrolled Medicaid patients with medical and mental health comorbidities  11,105 medicaid patients treated in CCI practices | low SES | CCI – a model of care that includes behavioural health training, integrated depression screening and coaching to ensure coordination of behavioural and medical care.  Setting - Patient-centred medical homes, USA | Control group treated at comparison non-CCI practices propensity matched at patient level and clustering at practice level | - Changes in total per-patient costs | US Dollars |
| Romeo 2009 | To examine the effects of health check interventions to identify needs in adults with intellectual disabilities on service use and cost and hence how affordable such interventions are. | Analysis of service use patterns and costs over 12 months | The first 50 adults with intellectual disabilities to have the health check intervention | Adults with intellectual disability. | Health check intervention - includes semi-structured health assessment instrument reviewing general physical health, mental health, development and problem behaviours and selected physical examinations and blood tests.  Setting - UK | 50 matched control participants from a neighbouring population. | - Service use and costs | Pounds |

[TABLE 2 LEGEND]

CCI = chronic care initiative; CCS = Cultural consultation service; CD = conduct disorder; CYP = Children and young people; ODD = oppositional defiant disorder; PTSD = post-traumatic stress disorder;; SES = socioeconomic status

Table 3: Research question 3 primary studies

| **Study ID (first author, date)** | **Study aims** | **Study design** | **Population sample** | **Population characteristic/s associated with inequality** | **Intervention detail** | **Comparator** | **Outcomes**  **/Themes** |
| --- | --- | --- | --- | --- | --- | --- | --- |
| Adams 2015 | To evaluate the impact of removing a significant financial barrier to prescription medications (drug caps) on existing black-white disparities in antidepressant treatment rates among patients with diabetes and comorbid depression. | Interrupted time series to examine changes in the monthly rate of anti-depressant treatment 24 months before and after the transition of dual enrolees from Medicaid to Medicare Part D drug coverage. | 31 US states. Black and white dual enrolees of Medicare and Medicaid - a 5% nationally representative sample. Patients were 18+ with a diagnosis of diabetes. A subset of patients also had major depression. | Race: Black-white disparities | Removal of drug caps in Medicaid/ Medicare plans (financial barrier to prescription medication).  Setting -prescriptive settings in the USA. | States with no removal of drug cap. | Outcomes   - proportion of patients with any antidepressant use per month - mean standardised doses   Themes – n/a |
| Aggarwal 2016 | To examine how patients, clinicians, and administrators define the meanings and practices of cultural competence in one hospital; to: (1) compare themes within and across three focus groups conducted separately with patients, clinicians, and administrators, and (2) assess how these themes relate to cultural competence policies at the hospital study site. | Analysis of focus group data; focus groups stratified using theoretical sampling | A diverse group of patients receiving mental health treatment and clinicians and administrators using services working in the psychiatric department of a hospital. | Not specified: study reports on a 'diverse' community. | No intervention  Setting - psychiatry department in community hospital, USA | Compared focus group responses to hospital policies. | Themes –   - definitions of CCS - clinician techniques - patient challenges - clinician challenges - institutional changes |
| Allerton 2012 | To quantify the extent to which adults with chronic health conditions or impairments in Great Britain experience barriers to accessing health services in comparison to adults without impairments. | Secondary analysis of cross-sectional population-based survey data from the Life Opportunities Survey. | A nationally representative sample of 37,513 people aged 16+ from the Life Opportunities Survey. Proportion of sample with mental health/behavioural impairments was n=611 (2.3%). | Disability (unspecified). | No intervention.  Setting - health care settings not specified, UK. | Adults without impairments | Themes –   - physical - access - communication difficulties - discrimination by staff |
| Baba 2014 | To identify clients' self-identified health needs to inform the post-2015 international development goals. | Thematic analysis of transcripts from focus groups and semi-structured interviews. | Clients of both government-controlled Aboriginal Medical Services (AMS), which provide a culturally appropriate alternative to mainstream medical services, and Aboriginal community-controlled health service (ACCHS). | Aboriginal communities' health disparities. | AMS provides a culturally-appropriate alternative to mainstream care and ACCHS  Setting - community health settings, Australia. | n/a | Themes   - Addressing community-specific essential health needs - cultural identity - education - governance structures |
| Bogenschutz 2014 | To discover the particular challenges that immigrants with disabilities face when accessing health care, and the facilitating factors that assist them in this process. | Content analysis of interview data, qualitative study: complex multi-case study design. | Purposively sampled 9 individuals from 3 different immigrant communities (Hmong, Mexican and Somalian), having an array of developmental disabilities. | Immigrants; Disability (varied, physical and intellectual). | No intervention.  Setting - heath care settings not specified, USA. | n/a | Themes   - access to healthcare |
| Burra 2012 | To examine differences in services available at the time of discharge for homeless and housed psychiatric inpatients. | Cross-sectional study using survey and health record data: questionnaire and interview data, clinical and substance use scales, and data from electronic health records. | Thirty homeless people | Homelessness | No intervention  Setting - care in community following discharge from acute inpatient psychiatric unit, Canada. | 21 matched controls with schizophrenia/ schizoaffective disorder | Themes   - homelessness - housing - health care (general) - serious mental illness - inequalities for people with psychotic disorders - discharge from hospital |
| Chinn 2016 | To examine how the legitimacy of claims by people with intellectual disabilities to use this service is facilitated or impeded.  The study was looking at access to IAPT for people with ID and common mental health disorders. | Sequential mixed methods design: interview and survey data. | Interviews with people with intellectual disability (ID) and their carers  Online survey completed by 452 staff from IAPT and specialist ID services. | Intellectual/ learning disability. | IAPT (note, study is not looking at outcomes of IAPT or effect of intervention but access to IAPT care).  Setting - IAPT care, UK. | n/a | Themes   - process of referral to IAPT - delivery of treatment - training and service - development - suitability and effectiveness of IAPT for people with ID |
| Cristofalo 2009 | To capture the understudied and neglected perspective of providers in community health centres by describing frontline providers' attitudes, experiences, and concerns about the mental health and substance abuse problems of their patients, barriers to obtaining needed treatment, and proposed solutions to providing for this unmet need. | Content analysis of interview data. | Seventeen community health clinic providers and personnel | Race; socioeconomic disadvantage. | No intervention.  Setting - Community mental health care, USA. | n/a | Themes –   - complexity of patient needs - intraclinic and extraclinic systems - intra and extra clinic funding - social factors |
| Dalton 2018 | To analyse the characteristics of participants who did not complete the Health-e Babies pregnancy app pilot study requirements, in an attempt to identify potential barriers associated with the implementation of the app. | Retrospective review of qualitative and quantitative data from intervention trial commencement. | Women 18+, 10-14 weeks gestation, possession of an Android phone or tablet. | Pregnant women; low socio-economic status. | Health-e Babies App designed to provide health information about early pregnancy that would increase maternal confidence and reduce anxiety.  Setting - health care settings unspecified, Australia. | n/a | Outcomes –   - scores on various psychological assessment scales |
| De Silva 2017 | To explore GPs’ experience and views on which factors influence access to mental health services for mild to moderate depression in people living in rural areas. | Thematic analysis of interview data (general inductive method). | Interviews with ten GPs | Rural locations/communities | No intervention.  Setting - general practice settings, Australia. | n/a | Themes –   - management - referral patterns - barriers and enablers to management |
| Dewey 2015 | To investigate decision making among medical and therapeutic professionals who work with trans‐identified patients to understand factors that might impede or facilitate the adoption of the collaborative decision‐making model in their clinical work. | Analysis of semi-structured interview data using grounded theory approach. | 10 US physicians and 10 US mental health professionals | Trans-identified people. | Collaborative care model.  Setting - USA | n/a | Themes –   - collaborative care - treatment roles treatment dilemmas |
| Dowrick 2013 | To better understand dimensions of access and factors limiting access to psychological interventions for disadvantaged communities. | Mixed-methods study. Quasi-experimental design with 'no intervention' comparator to test a multifaceted model that was designed to improve access to mental health services in primary care. | Evidence from 7 sources used to inform a multifaceted model which was tested in 4 disadvantaged localities with focus on older adults and ethnic minority groups.  Intervention tested with 57 ethnic minority and older people | Disadvantaged localities; older people; minority ethnic populations. | Culturally-sensitive wellbeing intervention; interactive training packages.  Setting - mental health services in primary care, UK. | No comparator | Themes –   - equity of access - quality and responsiveness of services - problems with access:   Outcomes   - varied outcomes for each element of the study |
| Dowrick 2016 | To assess the effectiveness of a complex model in increasing access to and improving the quality of primary mental health care. | Multi-method study. Quasi experimental design with no intervention comparators to test the effectiveness of a previously developed model  Framework analysis used to analyse findings from qualitative data. | Model implemented in 4 underserved locations in North West England, focussing on older people and ethnic minorities.  Interview and focus group responses to topic guides from 110 participants | Disadvantaged localities; older people; minority ethnic populations. | Combined model of mental health in primary care (including culturally-sensitive wellbeing intervention).  Setting - mental health services in primary care, UK. | No comparator | Themes –   - access to psychological interventions - community awareness - professional agency - primary care quality - wellbeing and community engagement |
| Gibson 2011 | To explore the perspectives on tele-mental health of community members living in two rural and remote First Nations communities in Ontario, Canada. | Thematic analysis of qualitative interview data and descriptive quantitative analysis. Participatory research design. | Remote and rural First Nations communities using information and communication technologies. Interviews with 59 community members | Rural and remote communities. | Tele-mental health (note, study is not looking at outcomes of tele mental health but of opinions and attitudes and experiences).  Setting - Tele-mental health, Canada. |  | Themes –   - advantages of telemental health: usefulness, reductions in travel, client comfort/facilitation of disclosure. - Disadvantages/ concerns: appropriateness of using video conferencing, privacy and security issues, safety concerns; interference with capacity building; problems with technology, ambivalence/ uncertainty. |
| Glick 2016 | To examine the current ownership, usage patterns, and existing barriers to mobile health interventions for people with SMI treated in a public sector community mental health setting and to compare the findings with national usage patterns from the general population. | Logistic regression analysis of survey data.  Frequency distributions were used to describe barriers to smartphone ownership and interest in mobile mental health service | Surveys were administered to 100 patients with SMI at an outpatient psychiatric clinic. | Socioeconomic disadvantage (and SMI). | Mobile health interventions: mental health interventions offered through use of smartphone or mobile device.  Setting - Community mental health centre/outpatient clinic, USA. | Respondents to the 2012 Pew Survey of mobile phone usage. | Themes –   - mobile health - barriers to smartphone ownership - interest in mobile mental health service |
| Hailemariam 2016 | To inform delivery of a new primary care-based mental health service in rural Ethiopia by identifying potential barriers to equitable access to mental healthcare and strategies to overcome them. | Framework analyses of interview and focus group data. | Interviews with 21 stakeholders selected purposefully from mental health service users, caregivers, community leaders and health administrators. Focus group discussion n=12 conducted with community health extension workers. | Rural communities | Programme for improving Mental healthcare (PRIME): project supporting delivery of mental health care integrated into primary care in rural district.  Setting - Primary care-based mental health care, Ethiopia. | n/a | Themes -   - availability - affordability and accessibility - acceptability and adequacy - equitable access |
| Hepworth 2016 | To examine the impact of an innovation to integrate mental health care into primary care settings by employing a psychologist and a social worker. | Mixed-methods design: qualitative open-ended interviews with clients and focus groups with health workers; quantitative data pertaining to occasions of service. | Interviews with 7 psychology clients, 5 social work clients, the practice dietician, and the social worker and psychologist.  4 focus groups with GPs, practice nurses, Aboriginal Health Workers and receptionists | Aboriginal and Torres Strait Islander people. | Integrated mental health and primary care.  Setting - primary care, Australia. | n/a | Themes –   - responsiveness to community needs - trusted relationships shared cultural backgrounds and understanding |
| Hess 2014 | To examine the Refugee Wellbeing Project (RWP) programme that helps improve mental health of refugees in the US. | Analysis of historical qualitative structured interview data. | 53 student-refugee paired interviews and 24 student-student paired interviews conducted with all refugee and student participants between 2007 and 2009. | Refugees. | RWP.  Setting - Mental health care unspecified, USA. | n/a | Themes –   - experiences of transformative learning - transformations of self - humanising the 'other' - recognition of social inequalities and relationship to disparities - desire to effect structural change and reorienting of future goals. |
| Kahalnik 2018 | To evaluate use of VitalSign6 program in improving primary care practices to detect major depressive disorder. | Analysis of semi-structured interview data. | Primary care providers (n=11) | Low income/socioeconomic status; Latino and migrant populations. | VitalSign6: A multi-faceted training program designed to build confidence and competence in clinics' staff and improve performance. Uses point-of-care, web-based, self-report-based software program to provide universal depression screening in primary care practices and assist providers in monitoring and treating patients' symptoms using principles of Measurement-Based Care.  Setting - primary care clinics, USA. | n/a | Themes –   - barriers to treatment - provider empowerment - quality care - clinic workflow |
| Knifton 2012 | To examine beliefs, stigma and the effectiveness of existing national mental health campaigns with Pakistani, Indian and Chinese heritage communities in Scotland, UK, using community based participatory research approach. | Systematic analysis of focus group data using grounded theory approach. | 10 focus groups with participants (n=87) using a range of languages. | Minority ethnic communities: Pakistani, Indian and Chinese communities. | National campaigns aimed to address mental health stigma.  Setting - mental health care unspecified, UK. | n/a | Themes –   - beliefs and stigma about mental illness - perceptions of mental health treatment and services - effectiveness of an existing national anti-stigma campaign - new approaches |
| Krause 2018 | To collect more information for understanding mental health care access and experiences of economically disadvantaged people by incorporating patients’ views. | Analysis of semi structured interview data using grounded theory approach. | Participants from Chile and Columbia (n=24, adults) | Economically disadvantaged groups. | No intervention.  Setting - Mental health care and psychotherapy, Chile and Colombia. | n/a | Themes –   - access and experience (contextual and institutional aspects, and patient characteristics) - therapeutic experience and outcomes |
| Leahy 2018 | To examine the role of GP in providing early intervention and treatment for youth mental health problems. | Mixed methods study: qualitative analysis of interviews and quantitative analysis of cross-sectional survey data. | Qualitative interviews with health-care workers from primary care, secondary care and community agencies (n = 37) and young people (n = 20) in two of Ireland's most socioeconomically disadvantaged areas and a national cross-sectional survey of GPs (n = 175). | Young people; socioeconomic  disadvantage. | No intervention.  Setting - primary care, secondary care, community agencies involved in mental health care, Ireland. | n/a | Themes –   - help-seeking - early intervention for mental health and substance use disorders - GP characteristics, - education and training - GP management of people with mental health substance use disorders - barriers to addressing mental health /substance use disorders - attitudes to treating youth mental health problems |
| McEvoy 2017 | To reflect upon the Eis Leader initiative which sought to improve uptake of NHS mental health services for the Orthodox Jewish community. | Mixed methods study: qualitative and quantitative data. | Naturally occurring data (observational notes, e-mail), routinely collected demographic data and clinical outcomes measures from the initiative.  Written feedback and recorded discussions with 12 key informants. | Minority ethnic community: Orthodox Jewish Community. | Setting - various (IAPT services, phone /written discussions/ feedback), UK. | n/a | Themes –   - establishing an arms-length relationship - building a collaborative partnership - building a mature collaborative partnership. |
| Memon 2016 | To determine perceived barriers to accessing mental health services among people from BAME populations to inform the development of effective and culturally appropriate services to improve equity in health care. | Thematic analysis of qualitative focus group data. | Two focus groups consisting of 26 adults from BAME backgrounds. | Race: BAME populations. | No intervention.  Setting - mental health care unspecified, UK. | n/a | Themes –   - access to mental health services related to personal and environmental factors, - factors affecting the relationship between service user and healthcare provider |
| Osok 2018 | To examine interpersonal, practical, and cultural challenges to accessing care faced by pregnant adolescents with mental health needs in Kenya. | Qualitative study design; grounded theory analysis of interview data | Twelve first-time pregnant adolescents (aged 15-19) screened as positive on PHQ-9, were interviewed. | Pregnant/expectant adolescents. | No intervention.  Setting - mental health care unspecified, Kenya. | n/a | Themes   - challenge areas: depression, anxiety and stress around pregnancy; denial of the pregnancy; lack of basic provisions and care; limited opportunities for personal development (educational or occupational) post-pregnancy |
| Owiti 2014 | To improve on patients’ outcomes and clinicians’ cultural competence skills. | Ethnographic interview method. | Targeted 94 clinicians in 4 mental health service teams in the community. Provided initial training sessions and then used cultural consultation model to facilitate learning. | Race/ethnicity; diverse communities. | Cultural consultation service (CSS): an innovative model to promote cultural competence of clinicians.  Setting - community health services unspecified, UK. | n/a | Themes   - cultural competence |
| Platell 2017 | To identify barriers and enablers to professional mental health service utilisation for disadvantaged adolescents from the perspective of mental health service providers. | Content analysis of semi-structured interview data. | Eight semi-structured, face-to-face interviews completed with purposively recruited government, non-government and school mental health service | Disadvantaged adolescents/young people. | No intervention.  Setting - mental healthcare services unspecified, Australia. | n/a | Themes   - pathways to mental health services - how services listen to and interact with adolescents - service environment including youth-friendly locations and atmosphere - government and organisational policies |
| Posselt 2017 | To investigate the barriers and facilitators to culturally responsive comorbidity care for young refugees and to assess whether the mental health and alcohol and other drug (OAD) services were equipped to provide adequate support. | Mixed-methods study: thematic analysis of qualitative interview data and quant online survey data. | Interviews with 15 refugee youth and 15 service providers  Online survey with 56 managers of services. 56 (40 complete, 16 partially-complete) | Refugees (with comorbid mental health and substance use). | No intervention  Setting - mental health and substance use services, Australia. | n/a | Themes –   - organisational and structural barriers - access and engagement - treatment and service delivery - training and resources |
| Singh 2013 | Study 1: to understand ethnic differences in pathways to care in FEP by exploring cultural determinants of illness recognition, attribution and help-seeking among different ethnic groups. Study 2: to evaluate the process of detention under the Mental Health Act (MHA) and determine predictors of detention. Study 3: to determine the appropriateness, accessibility and acceptability of generic early intervention services for different ethnic groups. | Mixed methods study (one paper comprising of 3 separate studies, each with a qual and quant component). | Study 1: Prospective cohort of FEP patients and their carers recruited over a 2-year period . A stratified subsample of user–carer interviews were subjected to qualitative analyses  Study 2: Clinical and sociodemographic data for all MHA assessments conducted between April 2009–March 2010. 5 cases from each major ethnic group were randomly selected for a qualitative exploration of carer perceptions.  Study 3: Focus groups with service users, carers, health professionals, key stakeholders from voluntary sector and community groups, commissioners and representatives of spiritual care | Race: BAME populations. | No intervention.  Setting - mental health care unspecified, UK. | n/a | Themes –   - pathways to mental health care for BAME groups   Outcomes –   - Mental Health Act assessments |
| Wallin 2016 | To evaluate the preference for Internet-based psychological interventions as compared to treatment delivered face to face among individuals without past or current use of mental health treatment delivered online; and to investigate predictors of treatment preference and to complement the quantitative analyses with qualitative data about the perceived advantages and disadvantages of Internet-based interventions. | Mixed methods study: qualitative analysis of survey data, quantitative analysis of predictors of treatment preference. | Two convenience samples were used. Sample 1 was recruited in an occupational setting (n=231) and Sample 2 consisted of individuals previously treated for cancer (n=208). | 'Far-reaching' populations | Internet-based interventions.  Setting - online mental health treatment, Sweden. | n/a | Themes –   - face-to-to face vs. online treatment advantages and disadvantages |
| Weich 2012 | To explore service users' and carers' accounts of recent episodes of severe mental illness and of the care received in a multi-cultural inner city. To examine factors impacting on these experiences, including whether and how users and carers felt that their experiences were mediated by ethnicity | Qualitative interview study. | Forty service users and thirteen carers were recruited following an acute psychotic episode using typical case sampling. | Race/Ethnicity: BAME populations. | No intervention.  Setting - acute mental health care settings, UK. | n/a | Themes –   - perspectives on difficulties - contribution of family and friends - experiences of acute care |
| Zarnowiecki 2018 | To describe the complexity of Program clients in the Central Australian family partnership program, understand how client complexity affects program delivery and the implications for desirable program modification. | Mixed methods study: quantitative data from the programme and qualitative data collected from semi structured interviews. | Australian Nurse-Family Partnership Program data collected using standardised data forms by nurses during pregnancy home visits (n=276 clients from 2009 to 2015) were used to describe client complexity and adversity in relation to demographic and economic characteristics, mental health and personal safety. Semi-structured interviews with 11 Australian Nurse Family Partnership Program staff and key stakeholders explored in more depth the nature of client adversity and how this affected Program delivery. | Aboriginal mothers and infants. | The Australian Nurse-Family Partnership Program.  Setting - post-natal care, Australia | n/a | Themes –   - adversity and complexity affect the delivery of the programme |

[TABLE 3 LEGEND]

ACCHS = Aboriginal community-controlled health service; AMS = Aboriginal Medical Services; BAME = Black, Asian and minority ethnic; CCS = Cultural consultation service; FEP = first episode psychosis; GP = General Practitioner; IAPT: Improving Access to Psychological Therapies; ID – intellectual disability; MHA = Mental Health Act; n/a – not applicable; NHS = National Health Service; OAD = alcohol and other drug; PRIME = programme for improving mental health care; PTSD = post-traumatic stress disorder; RWP = Refugee Wellbeing Project; SMI = serious mental illness
