# Supplementary material for: Advancing mental health equality: a mapping review of interventions, economic evaluations and barriers and facilitators
Source: Syst Rev. 2020 May 26;9:115. doi: 10.1186/s13643-020-01333-6 (PMC7251669; doi:10.1186/s13643-020-01333-6)
Supplement: Supplementary file 3 — Additional file 3. Tabulated study characteristics for included systematic reviews. [file 13643_2020_1333_MOESM3_ESM.docx]

Additional file 3: Study characteristics of systematic reviews for research questions 1 and 3

Research question 1: what studies are there on interventions to address or reduce mental health inequalities?

Research question 3: what are the barriers and facilitators to interventions to address or reduce mental health inequalities?

Table 1: Research question 1, systematic reviews

| **Study ID (first author + year)** | **Study aims** | **Included studies** | **Population characteristic(s) associated with inequality** | **Intervention details** | **Intervention types and strategies** | **Comparator(s)** | **Outcomes** | **Conclusions made by study author(s)** |
| --- | --- | --- | --- | --- | --- | --- | --- | --- |
| Bhui 2015 | To review the evidence on interventions designed to improve therapeutic communications between Black and minority ethnic patients and clinicians who provide care in psychiatric services. | 21 studies:   - 12 RCT - 2 non-randomised and comparative observational studies - 3 consecutive case series - 2 qualitative - 2 case studies | Minority ethnicities | Interventions to improve therapeutic communications between BAME patients and professionals in psychiatric services. | Types – Access, Intervention    Strategies – EC, OCA, OT | (as applicable) Treatment as usual, non-adapted treatment | - Improved therapeutic communication - Clinical symptoms of anxiety, depression of psychosis - Insight; impairment; functioning; adherence - Patient experience; patient satisfaction; knowledge/attitude towards treatment; stigma | Both culturally adapted psychotherapies and ethnographic and motivational assessment leading to psychotherapies were effective and received well by patients and carers. More research is needed from locations other than the UK and USA, including economic evaluations. |
| Garcia 2018 | To investigate the effectiveness of the collaborative care model for depression for LEP (Limited English Proficiency) patients in primary care. | 15 papers representing 9 studies:   - 5 RCTs - 3 cohort studies - 1 case control which compared collaborative care to usual care. | People with depression and limited English-speaking proficiency | Collaborative care for depression in adults with LEP in primary care. | Types – Access  Strategies - RSCT, ELLS, OCA | Usual care | - Depressive symptoms | Collaborative care for depression delivered by bilingual providers may be more effective than usual care for patients with LEP. |
| Gardner 2017 | To pool individual-level data from the IY parenting trials in Europe to examine to what extent it benefits socially disadvantaged families. | Individual participant data meta-analysis of 14 randomised trials of the IY parenting intervention | Children aged 2-10 years from socially disadvantaged families (included differential effects for ethnic minorities) | Incredible Years (IY) Parenting Program. | Types – Early intervention, Intervention  Strategies – DET, PPS | Families of differing SES, ethnicities; parenting styles and social advantage | - Disruptive child behaviour - Parenting practices; parenting stress and mental health - Child ADHD and emotional symptoms | Benefits of the IY intervention are greater in the most distressed families. The intervention may have effects in reducing inequalities due to parent depression. |
| Lucas 2008 | To assess the effectiveness of direct provision of additional monies to socially or economically disadvantaged families in improving children's health, well-being and educational attainment. | 9 RCTs including more than 25,000 participants | Pregnant women and families with children, with low socio-economic characteristics | Providing money | Types – Prevention, access  Strategy - PFIP | No-intervention controls (3 studies also included alternative intervention groups) | - Child health - Child mental health and emotional state - Child cognitive development, education and achievement | Current evidence does now allow for the statement that financial benefits interventions are unequivocally effective in improving child health or wellbeing in the short term. |
| Pega 2017 | To assess the effects of UCTs for improving health services use and health outcomes in vulnerable children and adults in LMICs. To assess the effects of UCTs on social determinants of health and healthcare expenditure and to compare to effects of UCTs versus CCTs. | 21 studies:   - 16 cluster-RCTs - 4 CBAs - 1 cohort study involving 1,092,877 participants (36,068 children and 1,056,809 adults) and 31,865 households in Africa, the Americas and South-East Asia | Children and adults with low socio-economic characteristics from low and middle-income countries | Unconditional cash transfers (UCTs) | Types – prevention,  access  Strategy - PFIP | CCT interventions, no CTT or smaller UCTs | - Health service use - Any health outcome (including mental health, such as depression) | While UCTs may not impact a summary measure of health service use in children and adults in LMICs, they may improve some health outcomes (such as likelihood of having had any illness, food security and level of dietary diversity), one social determinant of health (likelihood of attending school) and healthcare expenditure. Evidence on relative effectiveness of UCTs and CCTs is uncertain. |
| Rojas-Garcia 2014 | To identify, characterize, and analyse the effectiveness of healthcare interventions for perinatal depression in socially disadvantaged women. | 16 studies (RCTs with a combined total of 1,647 participants. | Minority ethnicity mothers with low socio-economic characteristics | Interventions to decrease depressive symptoms in socially disadvantaged women e.g. Home-Start UK, ROSE program, Living in Harmony program, IPT, psychoeducational, Mothers and Babies course and CBT courses | Types – early intervention, intervention  Strategies - DET, PPS, OCA | Usual care or ‘other intervention’ | - Depressive symptoms - Social adjustment or social support | Healthcare interventions specifically targeted to low-SES women with perinatal depression can significantly reduce symptoms of depression. Culturally adapted interventions that are individually administered are more likely to succeed. |
| Vallury 2015 | To systematically review the global evidence regarding the clinical effectiveness and acceptability of CCBT interventions for anxiety and/or depression for people living in rural and remote locations. | 11 studies;   - 4 RCTs - 3 quasi-experimental, - 1 systematic review - 2 survey or qualitative - 1uncontrolled trial | People living in rural or remote communities | Computerised CBT (CCBT) | Types – access, intervention  Strategies - PPS, OT | People living in urban communities | - Acceptability of interventions - Depression, anxiety and quality of life | CCBT can be effective in treating depression and anxiety and is generally acceptable among people living in rural/remote locations. More work is needed to determine the most feasible model of CCBT delivery. |
| van der Waerden 2011 | To provide an overview of controlled outcome studies and to investigate the overall efficacy and moderators of interventions targeted at reducing depressive symptoms in women with low SES. | 14 studies including a total of 1,396 participants.   - 13 RCTs - 1 non-randomised study | Women with low-socio-economic characteristics. | Various: 6 = prevention (e.g. psychoeducation, stress management or counselling), 4 = inter-personal therapy, 3 = social support.  Of these, 5 = in home or telephone, remainder were group-based | Types - early intervention,  intervention  Strategies - DET, PPS, OCA | Treatment as usual or a different intervention | - Symptoms and/or presence of depression | On average, programs developed specifically for low-SES women have been found to reduce depressive symptoms, indicating that mental health benefits can be obtained amongst this population. |
| Weaver 2018 | To describe known empirical studies on the topic of mental health Community Health Workers (CHWs), assess their effectiveness and methodological rigor, highlight CHW roles, and suggest directions for future research. | 9 studies including a total of 1,330 participants.   - 5 RCTs - 4 pre-post pilot studies | Disadvantaged communities including ethnic minorities and immigrants, people of low socio-economic status, gender (both male and female), pregnancy and location. | Community Mental Health Worker supported interventions, particularly for addressing mental health needs in disadvantaged populations | Type – Access  Strategies – EC, OCA | Usual care or a different intervention (some included studies did not have a control group i.e. pre-post interventions) | - Depression, general mood and alcohol use | CMH-supported mental health interventions show promise and there is evidence of their feasibility and acceptability among underserved populations. |

[TABLE 1 LEGEND]

ATSI = Aboriginal or Torres Strait Islander; BAME = Black, Asian or other minority ethnic; CALD = culturally and linguistically diverse; CBA = controlled before and after study; CBT = cognitive behavioural therapy; cCBT = computerised cognitive behavioural therapy; CCT = conditional cash transfers; CHW = community health worker; DET = delivering education and training; EC = engaging the community; EU = European Union; IY = Incredible Years; LEP = limited English proficiency; LGBTQI = lesbian, gay, bisexual, transgender, queer, or intersex; LMIC = low and middle income countries; OCA = other-cultural adaptation; OT = other- technology; PFIP = Providing financial incentives or removing financial barriers; PPS = providing psychological support; RCT = Randomized controlled trial; RSCT = restructuring the care team; SES = socioeconomic status; UCT = unconditional cash transfers

Table 2: Research question 3, systematic reviews

| **Study ID (first author, date)** | **Study aims** | **Included studies** | **Population characteristic(s) associated with inequality** | **Outcomes/themes** | **Conclusions made by the author(s)** |
| --- | --- | --- | --- | --- | --- |
| Brown 2016 | To review the literature on barriers and facilitators to accessing and engaging with mental health care among young people from potentially disadvantaged groups. | 62 studies   - 24 qualitative studies - 38 quantitative studies | 'At risk' young people: ATSI, LGBTQI, CALD, homeless, substance users, rural/remote locations. | Themes - Access and engagement with health services; awareness of services; stigma and shame; motivation for treatment; belief systems with regard to seeking help; fear of unmet need; informal support outside of services | Young people who belong to two or more groups considered ‘at risk’ experience additional barriers to mental health care which may lead to increased disadvantage. Further, good quality research is needed to gain a better understanding of barriers and facilitators to accessing and engaging with mental health services among young, disadvantaged people. |
| De Vito 2016 | To address undocumented migrants' entitlement and barriers to healthcare using evidence from peer-reviewed and grey literature; people living in the EU without the legal right to remain/be in the destination country. | 54 studies which focussed on entitlement, barriers and policy. | Undocumented migrants. | Themes - Healthcare entitlement and barriers; socio-cultural barriers (language and communication); knowledge about the healthcare system; availability of formal and informal social and healthcare networks; providing appropriate care; cultural barriers; communication strategies; staff training needs; access. | Communication strategies play a key role in working to address inequalities in access to health care for undocumented migrants. The definition and provision of specific training aimed at meeting needs of undocumented migrants is desirable. |
| Derr 2016 | To synthesise current findings on mental health service utilisation among immigrants to inform future research efforts addressing disparities in access to care. | 62 studies   - 53 quantitative - 6 qualitative - 3 mixed-method   All were cross-sectional. | Immigrants. | Themes - Rates of mental health service use; service use patterns; provider preferences; barriers to service use; factors associated with service use | Structural barriers to service use included lack of insurance, high cost of care and language barriers. Social support may be particularly important for immigrants who, when they seek help, tend to first turn to family, friends and religious leaders. Future research is needed to expand knowledge on disparities in mental health service use among immigrants, especially to examine the role of social support in treatment engagement and foster collaboration between formal and informal sectors. |
| Lamb 2012 | To synthesise evidence relating to the access experiences of 'hard to reach' groups in England. | 20 qualitative studies | 'Hard to reach groups': the homeless, long-term unemployed, adolescents with eating disorders, depressed elderly people, advanced cancer sufferers, patients with medically unexplained symptoms, asylum seekers and people from black and minority ethnic groups. | Themes - Illness models; illness narratives; illness experience; stigma; self-management and coping strategies; disclosure; primary care experiences; perceptions of help-seeking | There are distinct difficulties involved when engaging ‘hard to reach’ groups through primary care. The reasons behind why many individuals do not approach primary care with mental health concerns must be addressed through raising awareness of the range of problems that primary care can provide support with and through the provision of signposts to more effective forms of self-management and coping. |

[TABLE 2 LEGEND]

ATSI = Aboriginal or Torres Strait Islander; CALD = culturally and linguistically diverse; EU = European Union; LGBTQI = lesbian, gay, bisexual, transgender, queer, or intersex
